# Uranium(III)-carbon multiple bonding supported by arene δ-bonding in mixed-valence hexauranium nanometre-scale rings

**DOI:** 10.1038/s41467-018-04560-7

**Published:** 2018-05-29

**Authors:** Ashley J. Wooles, David P. Mills, Floriana Tuna, Eric J. L. McInnes, Gareth T. W. Law, Adam J. Fuller, Felipe Kremer, Mark Ridgway, William Lewis, Laura Gagliardi, Bess Vlaisavljevich, Stephen T. Liddle

**Affiliations:** 1 School of Chemistry, The University of Manchester, Oxford Road, Manchester, M13 9PL UK; 2 School of Chemistry and Photon Science Institute, The University of Manchester, Oxford Road, Manchester, M13 9PL UK; 3 Department of Electronic Materials Engineering, Research School of Physics and Engineering, The Australian National University, Canberra, ACT 2601 Australia; 4 School of Chemistry, University Park, University of Nottingham, Nottingham, NG7 2RD UK; 5 Department of Chemistry, Supercomputing Institute and Chemical Theory Center, University of Minnesota, 207 Pleasant Street SE, Minneapolis, MN 55455 USA; 6 Present Address: Department of Chemistry, University of South Dakota, 414 E Clark Street, Vermillion, SD 57069 USA

## Abstract

Despite the fact that non-aqueous uranium chemistry is over 60 years old, most polarised-covalent uranium-element multiple bonds involve formal uranium oxidation states IV, V, and VI. The paucity of uranium(III) congeners is because, in common with metal-ligand multiple bonding generally, such linkages involve strongly donating, charge-loaded ligands that bind best to electron-poor metals and inherently promote disproportionation of uranium(III). Here, we report the synthesis of hexauranium-methanediide nanometre-scale rings. Combined experimental and computational studies suggest overall the presence of formal uranium(III) and (IV) ions, though electron delocalisation in this Kramers system cannot be definitively ruled out, and the resulting polarised-covalent U = C bonds are supported by iodide and δ-bonded arene bridges. The arenes provide reservoirs that accommodate charge, thus avoiding inter-electronic repulsion that would destabilise these low oxidation state metal-ligand multiple bonds. Using arenes as electronic buffers could constitute a general synthetic strategy by which to stabilise otherwise inherently unstable metal-ligand linkages.

## Abstract (web summary)

Owing to the propensity for uranium(III) compounds to undergo disproportionation, uranium-element multiple bonds involving uranium(III) oxidation states remain rare. Here the authors report hexauranium-methanediide rings that formally contain uranium(III)- and uranium(IV)-methanediides supported by alternating halide and arene bridges.

## Introduction

In recent years, motivated by a need to develop better extractants to reduce the volume of fission waste products in nuclear fuel cycles^1^, there has been sustained interest in probing the chemical bonding in uranium-element linkages^2–8^. Thus, once limited to uranyl(VI) chemistry, polarised-covalent uranium-element multiple bonding has flourished in response to the above need, to now include carbenes, imides and nitrides, phosphinidenes, arsinidenes and arsenidos, mono-oxos and heavier chalcogenidos^9–13^. However, although polarised-covalent uranium-element multiple bonds are now relatively common, they mostly encompass the IV, V, and VI oxidation states of uranium^14^ even though non-aqueous uranium research is over 60 years old. This situation is perhaps not surprising because polarised-covalent metal-ligand multiple bonds employ charge- and electron-loaded ligands that favour high oxidation state metal ions, and low oxidation state uranium(III) is prone to disproportionation in the presence of strong donor ligands and typically engages in ionic, lanthanide-like bonding that is ill-suited to covalent metal-ligand multiple bonding. This is fundamentally different to amide (R_2_N^−^) and alkoxide (RO^−^) complexes, where covalent/dative bonding combinations may occur, or *N-*heterocyclic/mesoionic carbenes, CO, N_2_, and NO complexes, where the uranium(III)-element bonding is of dative donor–acceptor character^15–21^.

Transition metal carbenes with polarised-covalent M = C double bonds have been known for decades, but it was not until 1981 that the first U = C double bond was isolated in the uranium(IV)–carbene complex [U(*η*^5^-C_5_H_5_)_3_{C(H)PMe_2_Ph}]^22^. After initial advances^23–26^, U = C double bond chemistry fell dormant for nearly 30 years with only occasional matrix isolation and reactive-intermediate contributions to the area^27–35^. The area was rejuvenated from 2009 onwards^36–54^, principally using pincer carbene-like methanediide ligands such as BIPM^R^ [BIPM^R^ = C(PPh_2_NR)_2_; R = SiMe_3_ (TMS), 2,4,6-Me_3_C_6_H_2_ (Mes), 2,6-Pr^i^_2_C_6_H_3_ (Dipp)] or SCS [SCS = C(PPh_2_S)_2_], and now includes derivatives where uranium is in oxidation states IV, V, and VI, which has enabled a better understanding of the nature of U = C double bonds. There are no polarised-covalent uranium(III)-carbon double bond derivatives^47,50^, which likely reflects a paucity of synthetic approaches and that pincer methanediides are relatively hard, formally dianionic ligands that stabilise high, not low, oxidation state metal centres. Indeed, when uranium(III)-precursors have been used with BIPM^R^ transfer reagents, disproportionation to elemental uranium(0) and uranium(IV)-complexes occurs^38^. This contrasts to rare earth BIPM^R^ congeners where the pincer formulation enforces metal(III)-methanediide interactions, but those trivalent metals do not disproportionate^55^. Thus, although these methanediides are very effective at stabilising mid and high oxidation state uranium ions, they are conversely ill-disposed towards preparing uranium(III)-derivatives, and so the synthesis of a polarised-covalent uranium(III)-carbon double bond presents an inherent and unmet challenge.

During the dormancy of covalent uranium-carbene chemistry, inverted sandwich diuranium–arene complexes emerged. The first examples of such complexes were [{U(RNC_6_H_3_-3,5-Me_2_)_2_}_2_(*μ*:*η*^6^-*η*^6^-C_6_H_5_Me)] (R = Bu^t^ or adamantyl)^56,57^, and then [{U(C_5_Me_5_)_2_}_2_(*μ*-*η*^6^:*η*^6^-C_6_H_6_)]^58^, and subsequently the area developed with over 40 reported variants of the general form L_*n*_U(*μ*-arene)UL_*n*_ (L = anionic ligand, i.e. amide, cyclopentadienyl, halide, BIPM^R^H)^59^. In a wider context, arene ligands have proven their ability to support low oxidation state metal ions including even formal uranium(II)^60,61^. Two classes of inverted sandwich diuranium complexes have emerged, L_2_U(*μ*-arene)UL_2_ complexes containing C_6_-arene dianions and uranium(III) ions, and L_3_U(*μ*-arene)UL_3_, complexes exhibiting C_6_-arene tetraanions and uranium(V) centres^59,62,63^. Despite the well-developed nature of the area, it is germane to note that all these complexes are of a ‘one-dimensional’ form L_*n*_U(*μ*-arene)UL_*n*_ with a maximum of two uranium ions, where the arenes act as electron reservoirs^59^.

Here, we report the synthesis and characterisation of hexauranium nanometre-scale rings that formally contain uranium(III)- and uranium(IV)-methanediides supported by alternating halide and arene bridges. Such a bonding arrangement is facilitated by a reservoir of δ-bonding to the arene bridges, thus circumventing the inherent mismatch of strong dianionic donor ligands to low oxidation state uranium(III) that would otherwise disproportionate. Thus, rather than the arene being the functional group focus, here it is overtly performing a role as an ancillary, facilitating ligand to stabilise another metal-ligand functional unit. These ‘two-dimensional’ polyuranium complexes also demonstrate that it is possible to go beyond the previous ‘one-dimensional’ diuranium–arene limit, perhaps paving the way to exploitation in the preparation of novel nanoscale magnetic assemblies^64^.

## Results

### Synthesis and exchange reactions

**Fig. 1:**
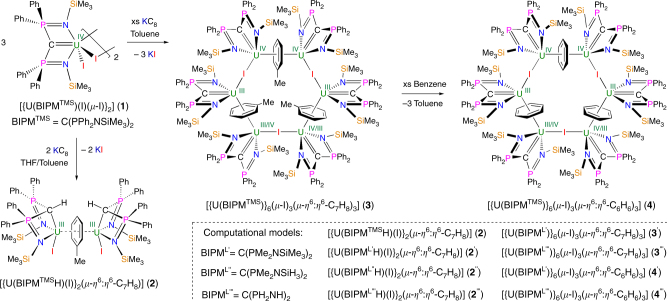
Synthesis of complexes **2**, **3**, and **4** along with definitions of computational models used in this study. See ref. ^43^ for the synthesis of complex **2**

Previously, we reported that reducing the uranium(IV) complex [{U(BIPM^TMS^)(*μ*-I)(I)}_2_] (**1**) with KC_8_ in toluene/THF gave the di(uranium(III)-methanide-iodide)arene complex, [{U(BIPM^TMS^H)(I)}_2_(*μ*-*η*^6^:*η*^6^-C_7_H_8_)] (**2**)^43^. The reaction is postulated to generate a uranium(III)-intermediate that deprotonates THF; we therefore envisaged that reducing **1** in the absence of THF might provide a route to uranium(III)-methanediides. Electrochemical interrogation of **1** in THF (see Supplementary Methods and Supplementary Fig. 1), unfortunately the only compatible solvent in which to electrochemically study **1**, reveals two irreversible reduction processes (*E*_P_^c^ = –0.72 V and –2.45 V vs. Fc/Fc^+^) accompanied by deposition of uranium(0). This indicates that reduction of this complex is realistic with alkali metals, though the putative uranium(III) product is not stable in that scenario and disproportionates to uranium(0) and uranium(IV) and/or attacks the THF solvent. Therefore, **1** was reduced with four equivalents of KC_8_ in toluene, and after work up the hexauranium ring complex [{U(BIPM^TMS^)}_6_(*μ*-I)_3_(*μ*-*η*^6^:*η*^6^-C_7_H_8_)_3_] (**3**), Fig. 1, was isolated.

As with other diuranium–arene complexes that contain formal uranium(III) ions^58^, we find that **3** undergoes toluene–benzene exchange to give [{U(BIPM^TMS^)}_6_(*μ*-I)_3_(*μ*-*η*^6^:*η*^6^-C_6_H_6_)_3_] (**4**), Fig. 1 and Supplementary Fig. 2. Complexes **3** and **4** crystallise from arene solutions in poor, but reproducible, yields of 12 and 1%, respectively, when prepared directly in either case. The crystalline products are only sparingly soluble in hydrocarbon and arene solvents; ~6 mg of **3** dissolves in 1 ml of hot D_6_-benzene, with the majority of the sample precipitating within 1 h, and **4** is even less soluble in D_6_-benzene (~1 mg in 1 ml). The addition of coordinating solvents to complexes **3** and **4** (e.g. Et_2_O, THF, MeCN, pyridine) causes decomposition.

### Solid-state structures

**Fig. 2:**
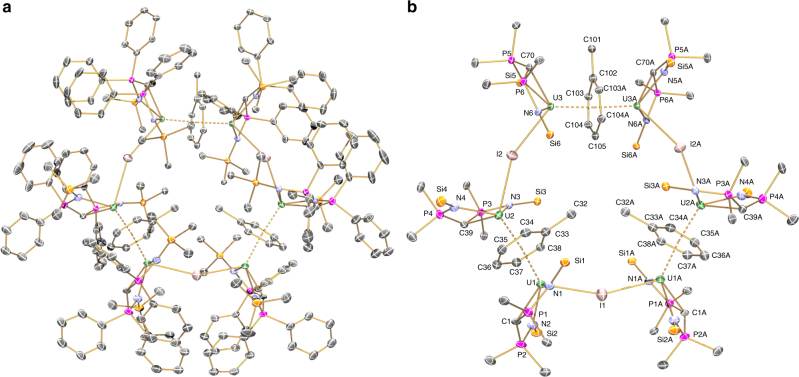
Molecular structure of **3** at 90 K and displacement ellipsoids set to 40%. **a** Full structure with hydrogen atoms, lattice solvent, and the disordered Ph_2_PCPPh_2_ portion of the BIPM^TMS^ coordinated to U1/U1A are omitted for clarity. **b** Structure highlighting the U_6_(*μ*-*η*^6^:*η*^6^-C_6_H_5_Me)_3_(*μ*-I)_3_ core with hydrogen atoms, lattice solvent, silyl-methyls, disordered *P*-phenyls bar the *ipso*-carbon, and the disordered Ph_2_PCPPh_2_ portion of the BIPM^TMS^ coordinated to U1/U1A are omitted for clarity. Each toluene methyl group is disordered such that they are directed towards the centre of the ring or in the opposite direction outwards and the combinations shown are arbitrary. Key: uranium, green; phosphorus, magenta; silicon, orange; iodide, pink; nitrogen, blue; carbon, grey

The solid-state structures of **3·**6C_7_H_8_ and **4·**26C_6_H_6_ were probed by single crystal X-ray diffraction, Fig. 2a and b and Table 1. These complexes are very similar, so only complex **3** is depicted and discussed (see Supplementary Fig. 3 for the structure of **4·**26C_6_H_6_). Complex **3** crystallises as a hexauranium ring with a diameter of ~2.5 nm. The ring topology is constructed from six uranium ions bridged by alternating arene and iodide ligands, with the remaining coordination sphere of each uranium ion completed by a BIPM^TMS^ ligand. The rings adopt extended chair-type conformations, where U(1), U(1A), U(3), and U(3A) are essentially co-planar, and U(2) and U(2A) each reside 2.108(6) Å above and below the mean plane.

**Table 1:** Selected bond lengths for **3** (Å)

U1^III/IV^-distances		U2^III^-distances		U3^IV^-distances	
U1–I1	3.0917(4)	U2–I2	3.1453(7)	U3–I2	3.1827(7)
U1–C1/–C1A	2.47(2)/2.30(3)	U2–C39	2.413(8)	U3–C70	2.398(7)
U1–C33	2.717(8)	U2–C33	2.718(8)	U3–C102	2.629(6)
U1–C34	2.678(9)	U2–C34	2.670(9)	U3–C103	2.590(8)
U1–C35	2.579(9)	U2–C35	2.647(8)	U3–C103A	2.669(8)
U1–C36	2.608(9)	U2–C36	2.602(9)	U3–C104	2.679(8)
U1–C37	2.653(9)	U2–C37	2.576(9)	U3–C104A	2.653(8)
U1–C38	2.674(8)	U2–C38	2.664(8)	U3–C105	2.863(7)

The oxidation states are formal and provided only as a guide

The BIPM^TMS^ ligands in **3** adopt open book^55^ configurations with short U = C distances [**3**: range 2.30(3)–2.47(2) Å] that correlate with the trend observed in previously reported uranium–BIPM^TMS^ complexes: uranium(IV)–[{U(BIPM^TMS^)(µ-Cl)(Cl)(THF)}_2_], U = C = 2.322(4) Å;^39^ uranium(V)–[U(BIPM^TMS^)(Cl)_2_(I)], U = C = 2.268(10) Å;^39^ uranium(VI)–[U(BIPM^TMS^)(Cl)_2_(O)], U = C = 2.184(3) Å^45^. These uranium–carbon distances confirm that the BIPM^TMS^ ligands in **3** and **4** are methanediides, in contrast to the methanides in **2** [U–C_methanide_ = 2.753(9) Å]^43^. Interestingly, the crystal structure of **3** hints that localised uranium(III) and uranium(IV) ions are present; U3 and U3A correspond to uranium(IV) centres [U = C = 2.398(7) Å], U2 and U2A are uranium(III) ions [U = C = 2.413(8) Å], and U1 and U1A correspond to a mixture of uranium(III/IV) ions with the BIPM^TMS^ ligand disordered over two locations [U = C = 2.30(3) and 2.47(2) Å]. This accounts for three uranium(III) and three uranium(IV) ions required for overall charge neutrality of **3**.

The *μ*-*η*^6^:*η*^6^-arene rings in **3** are approximately planar and exhibit a wide range of U–C distances [**3**: range 2.577(9)–2.723(9) Å], with mean U**···**U [**3**: 4.4466(5) Å] and U**···**arene_centroid_ [**3**: 2.224(4) Å] distances that are typical of uranium(III)–arene complexes, and these compare to corresponding values in **2** of 2.553(7)–2.616(7) (U–C), 4.2836(7) (U**···**U), and 2.142(4) (U**···**toluene_centroid_) Å^43^. The U**···**toluene_centroid_**···**U angles in **3** deviate little from linearity [174.6(2)–176.53(18)°] and there is no mirror symmetry through the arene plane unlike all other uranium arene complexes^59^, thus implicating the presence of U^III^-(*μ*-arene)-U^IV^ units in **3** and **4**.

The outer perimeter of the {U_6_I_3_(toluene)_3_} ring of **3** is completed by the bridging iodides [U–I–U angles range 154.84(3)–156.77(5)°]. Three crystallographically distinct, and revealing, U–I distances are observed in **3** [U1–I1, 3.0916(4); U2–I2, 3.1453(7); U3–I2 3.1826(7) Å]. The first value is associated with the disordered U^III/IV^-BIPM fragment and it also has the largest displacement ellipsoid in the structure so is not a reliable measure. However, we note that the shorter U2–I2 distance goes with a long U2–C39 bond length and conversely the long U3–I2 distance is associated with a short U3–C70 distance. The implication is that the methanediide ligand is the strongest donor and thus when it binds more closely the halide recedes.

### NMR and optical spectroscopies

The ^1^H NMR spectra of **3** and **4** in D_6_-benzene could be obtained. They are fully assignable and only minor protic impurities are observed, despite the low concentrations of **3** and **4** in D_6_-benzene. While the bulk features of the ^1^H NMR spectra of the BIPM^TMS^ ligands in complexes **3** and **4** are similar, they can be distinguished by small variations in their chemical shifts, with diagnostic, differing resonances of the bridging arene ligands (**3**: δ –1.35, –0.37, and 0.44; **4**: δ –0.25).

At 298 K no arene exchange of **3** or **4** with D_6_-benzene was observed by ^1^H NMR spectroscopy over 72 h. However, heating **3** in D_6_-benzene at 50 °C for 8 h gave **4**-D_6_ almost quantitatively, with formation of a small quantity of intractable by-products (see Supplementary Fig. 2). This reaction was monitored by ^1^H NMR spectroscopy, revealing that the reaction proceeds via at least two intermediates, which we suggest is the sequential replacement of toluenes by benzenes. Kinetic and thermodynamic parameters of this transformation could not be reliably elucidated due to variable precipitation of material during different reactions. Complex **4** was prepared by heating **3** in benzene at 50 °C for 8 h, and also by a competition experiment where **3** was heated in a mixture of D_6_-benzene and benzene (1:1) at 60 °C for 16 h, which gave **4** as the only soluble uranium-containing product. No reaction was observed at 60 °C between **3** and D_8_-toluene, or **4** with either D_6_-benzene or D_8_-toluene, and the use of elevated temperatures led to the formation of intractable products. The low solubilities of **3** and **4** in arene solvents limited the arene exchange experiments to the combinations above.

The electronic absorption spectrum of **3** was obtained (see Supplementary Fig. 4); the solubility of **4** in toluene is too low for a solution spectrum to be collected. In common with **2**, **3** dissolves in toluene to give intense brown solutions and its electronic absorption spectrum is dominated by a charge-transfer band trailing in from the ultraviolet region to ~9000 cm^–1^, which obscures any *f* → *d* transitions of uranium(III) that might be in the visible region of the spectrum. This charge-transfer band trails into the NIR region so that only one Laporte forbidden *f* → *f* transition was assigned (ύ = 11,600 cm^–1^, *ε*~400 M^–1^ cm^–1^) based on its similarity to an *f* → *f* transition observed for **2** (ύ = 11,100 cm^–1^, *ε*~600 M^–1^ cm^–1^)^43^. In the 3000–9000 cm^–1^ region, several absorptions characteristic of uranium(III) *f* → *f* transitions are observed for **3** (^4^*I*_11/2_: ύ = 4050 cm^–1^, *ε*~700 M^–1^ cm^–1^; ^4^*F*_3/2_: ύ = 7200 cm^–1^, *ε*~150 M^–1^ cm^–1^; ^4^*I*_13/2_: ύ = 8700 cm^–1^, *ε*~250 M^–1^ cm^–1^), which can be compared to **2** (^4^*I*_11/2_: ύ = 4130 cm^–1^, *ε*~4000 M^–1^cm^–1^; ^4^*F*_3/2_: ύ = 7070 cm^–1^, *ε*~900 M^–1^ cm^–1^; ^4^*I*_13/2_: ύ = 8370 cm^–1^, *ε*~1200 M^–1^ cm^–1^)^43^. The absorptions for **3** are approximately an order of magnitude less intense than **2**, but **3** contains proportionately half as many uranium(III) ions overall. Not all absorptions in this region could be assigned due to the complexity of the system, but this is a common feature of uranium(III) which can have, not including 5*f*–6*d* transitions, 182 crystal field sub-levels^14^. Notably, the optical spectrum of **3** is very similar to that of **2**; the latter by definition does not have inter-valence charge-transfer (IVCT) bands, and there is no evidence of IVCT bands for **3**.

### SQUID magnetometry and EPR spectroscopy

**Fig. 3:**
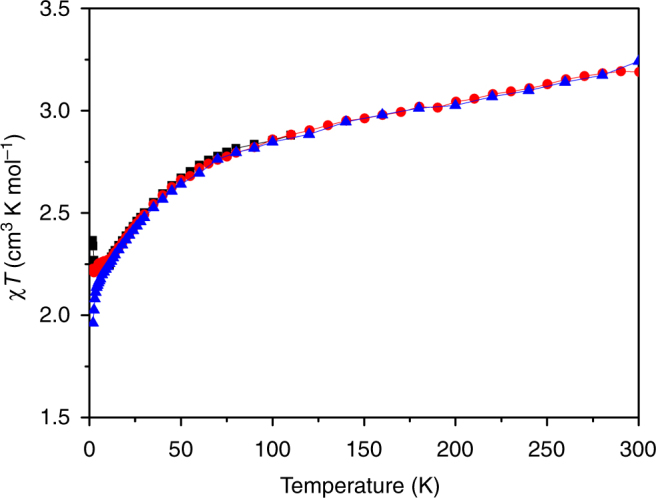
Variable temperature SQUID magnetometric data of powdered **3** in the solid state. Magnetic susceptibility temperature product (*χT*) vs. temperature in 1 (black square), 5 (red circle), and 10 (blue triangle) kG applied magnetic fields. Lines are a guide to the eye only

**Fig. 4:**
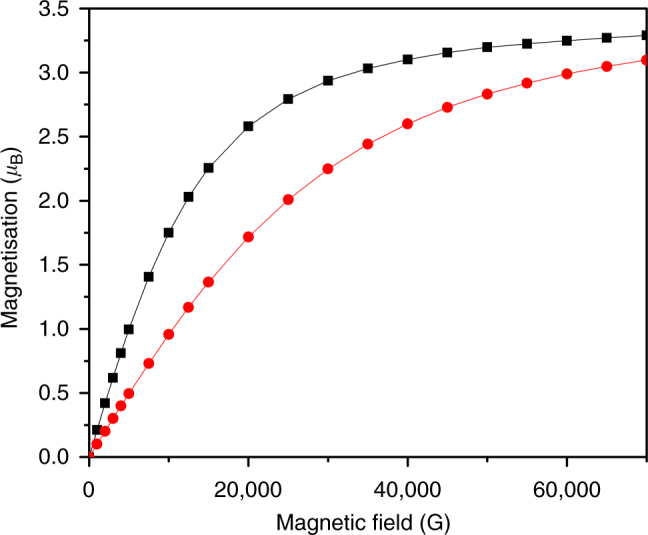
Static temperature SQUID magnetometric data of powdered **3** in the solid state. Molar magnetisation as a function of applied magnetic field at 4 (red dots) and 2 (black squares) K. Lines are a guide to the eye only

Uranium(III) and uranium(IV) are Kramers (^4^*I*_9/2_) and non-Kramers (^3^*H*_4_) ions, respectively. Because of this, uranium(IV) compounds are usually characterised by a *χT* product that tends towards zero at low temperature, where *χ* is the molar magnetic susceptibility, while that for uranium(III) will tend towards a finite value^13,43,65,66^. At room temperature, complex **3** has a *χT* of ca. 3.2 cm^3^ K mol^−1^ (which has not reached its high temperature limit), which then decreases on cooling reaching ca. 2.2 cm^3^ K mol^−1^ at 5 K. On further cooling, *χT* increases slightly to ca. 2.4 cm^3^ K mol^−1^ (for a 1 kG applied magnetic field; Fig. 3). The general decrease is due to thermal depopulation of the spin–orbit states of the uranium ions. The fact that the data do not tend towards zero suggests that some of the metal ions are uranium(III). This is corroborated by the easy saturation of magnetisation at low temperature, reaching ca. 3.3*μ*_B_ above 3–4 T at 2 K (Fig. 4).

**Fig. 5:**
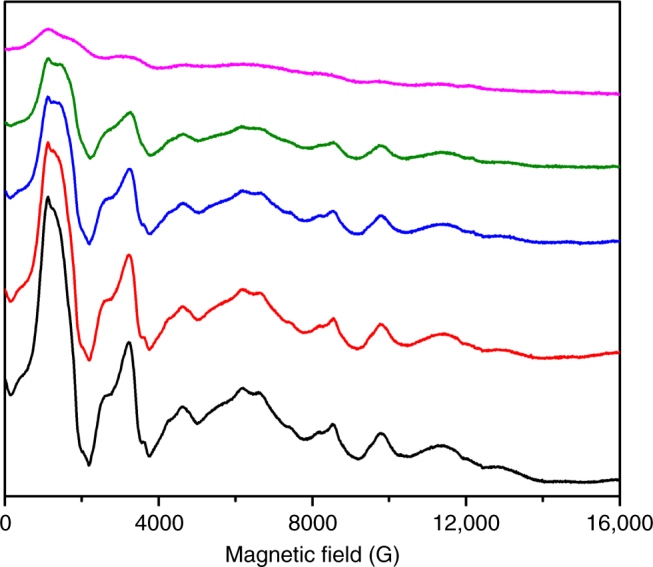
EPR spectroscopic properties of powdered **3** in the solid state. Variable temperature Q-band (34 GHz) EPR spectra at 30 (magenta), 20 (green), 15 (blue), 10 (red), and 7 (black) K

EPR spectra of **3** (observable below ca. 40 K) are unusually complex, with multiple resonances between 0 and 1.8 T at Q-band (34 GHz; Fig. 5). Since EPR is very sensitive to very weak interactions, the structure of the spectrum could be due to weak uranium–uranium interactions (peak separations of 0.1–0.2 kG, which corresponds to 0.1–0.2 cm^−1^), which could be consistent with the very low temperature rise in *χT*(*T*); a fully isolated uranium(III) ion typically gives a simple EPR spectrum characteristic of the effective *g*-values of the lowest energy Kramers doublet^66^. Quantitative analysis of the EPR and magnetic data is not possible at this stage given the complexity of the system. The large variations in typical magnetic moments of uranium(III) and uranium(IV) species preclude even a simple additive analysis; this is true even of smaller inverse-sandwich complexes, for example, **2** has *χT* of ca. 1.5 cm^3^ K mol^−1^ at room temperature^43^, significantly lower than that expected for the sum of two isolated uranium(III) ions (e.g. [U^III^(BIPM^TMS^H)(I)_2_(THF)] has *χT* ≈ 1.7 cm^3^ K mol^−1^ at room temperature)^43^. Nevertheless, even acknowledging that formal oxidation states starts to become a diffuse concept with covalently bonded uranium–arene fragments, the observation of EPR spectra at all for **3**, together with the magnetic data, are only consistent with a Kramers system.

### X-ray absorption near edge spectroscopy

**Fig. 6:**
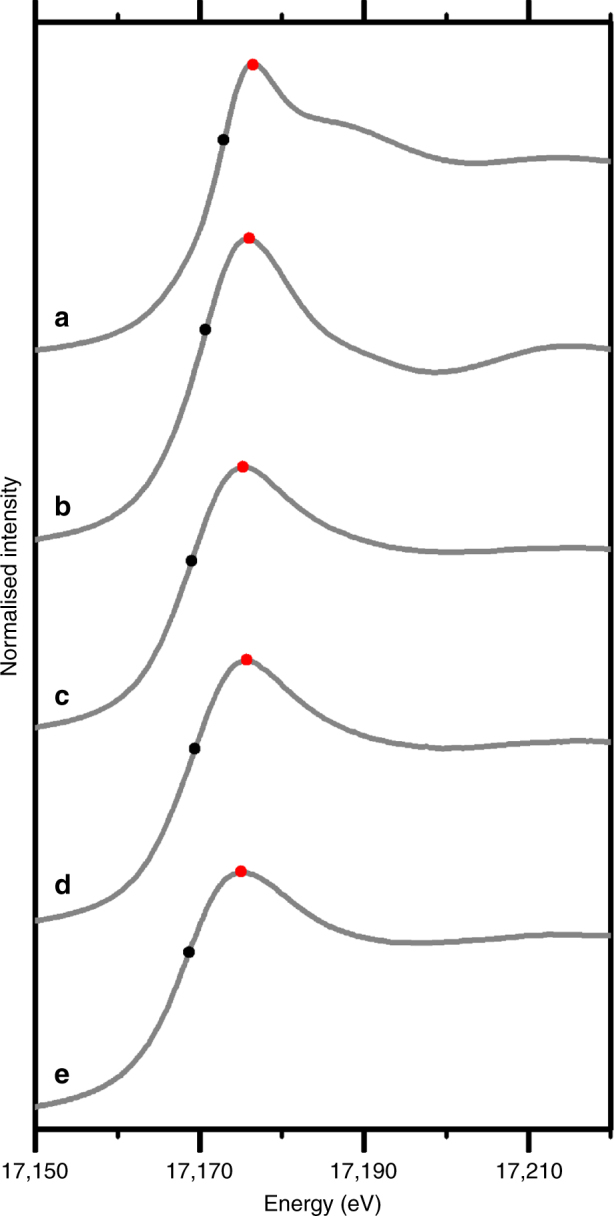
Uranium L_III_-edge XANES spectra. **a** U(VI)-standard, **b** U(IV)-standard, **c** complex **4**, **d** complex **3**, and **e** [UI_3_(THF)_4_]. Black dots mark the point where the second derivative of that XANES spectrum crosses zero (edge). Red dots indicate the position of the primal spectral XANES peak energy (white line) for that spectrum (where first derivative of the XANES trace crosses zero). The estimated uncertainty is ±0.2 eV

To further probe **3** and **4**, low-temperature uranium L_3_-edge X-ray absorption near edge spectroscopy (XANES) spectra were collected. Data were calibrated in energy space using a single oxidation state in-line reference foil. Further experimental details are given in the Supporting Information. The background-subtracted, normalised XANES of **3** and **4** are shown in Fig. 6 alongside data for uranium(III), uranium(IV), and uranium(VI) single oxidation state standards ([UI_3_(THF)_4_], UO_2_, and UO_2_^2+^ sorbed onto ferrihydrite, respectively). Samples were collected on two separate beam lines to ensure consistency. The sample spectra have strong white lines and are similar in shape to the UO_2_ and uranium(III) standards and other uranium(III) and uranium(IV) L_3_-edge XANES described in the literature^67–69^. Complexes **3** and **4** lack a shoulder feature on the high energy side of the white line indicating the absence of uranium(V) or uranium(VI), since a shoulder feature would be indicative of multiple scattering along the trans-dioxo moiety^67^.

We have used the zero-crossing point of the first derivative of the normalised XANES versus energy to define the primary spectral XANES white line energy for our complexes^67^, giving values of 17175.7 and 17175.3 eV for **3** and **4**, respectively. These energies are close to [UI_3_(THF)_4_] (17175.0 eV), are ~2 eV higher than those reported for uranium(III)-tacn complexes^67^, and ~1–2 eV lower than those recorded for UO_2_ (17176 eV in this study; 17177.2 eV in ref. ^67^) and a range of uranium(IV)-tacn complexes^67^. It should be noted that the energy separations of edge energies for organometallic species tends to be narrower than in inorganic compounds^68^. Another approach would be to use the point at which the second derivative of the normalised XANES is zero to assign an average uranium oxidation in similar complexes^68,69^. In that scenario the edge values for complexes **3**, **4**, and the [UI_3_(THF)_4_] and UO_2_ standards are now 17169.4, 17169.0, 17168.7, and 17170.7 eV, respectively. Equivalent literature values reported for other uranium(III) complexes range from 17167.0 to 17168.7 eV and 17169.8 to 17170.9 for uranium(IV) complexes and UO_2_^68,69^. Irrespective of whether the first- or second-derivative methods are used, the XANES data collected from **3** and **4** consistently suggest a mixture of uranium(III) and uranium(IV) ions in these complexes.

### Quantum chemical calculations

A well-established approach to study organometallic systems is to optimise the geometry with density functional theory (DFT) with subsequent single-point calculations using a high-level multi-reference method to study the electronic structure. While diuranium species such as **2** can be treated in this manner, compounds such as **3** and **4** demand deviations from this ‘standard’ approach due to their size and polymetallic nature. Therefore, we first studied **2** at varying levels of theory, including the complete active space self-consistent field (CASSCF) method, followed by second-order perturbation theory (CASPT2), and tested the effect that the truncations in the active space and the ligands have on the computed results; the ligand truncations involved sequential simplification using BIPM^L′^ = C(PMe_2_NSiMe_3_)_2_, BIPM^L″^ = C(PMe_2_NSiH_3_)_2_, or BIPM^L′′′^ = C(PH_2_NH)_2_, denoted as **2**′, **2**″, and **2**‴, respectively. The results for the largest model (complexes with BIPM^L′^ denoted **3**′ and **4**′) with DFT and results with the smallest model (complexes with BIPM^L′′′^ denoted as **2**‴ and **4**‴) with multi-reference methods are presented below while results for other models including computational details, testing of the level of theory, and a discussion of the active space are given in Supplementary Figs. 5–24, Supplementary Tables 1–14, Supplementary Methods, and Supplementary Note 1.

The electronic structure of the ring complexes were explored first with the restricted active space self-consistent field method (RASSCF) for the *S* = 7/2 to the *S* = 13/2 states. The active space in the RASSCF calculations is (21*e*, 2*e*, 2*e*; 6*o*, 11*o*, 6*o*) where the notation^70^ indicates a RAS space of (*n*, *l*, *m*; *i*, *j*, *k*) where *n* is the number of electrons in the active space, *l* is the maximum number of holes in RAS1, and *m* is the number of electrons allowed in RAS3. Similarly, *i*, *j*, and *k* are the number of orbitals in RAS1, RAS2, and RAS3 respectively. The *S* = 7/2 state of **4‴** is only 0.02 kcal mol^−1^ higher in energy than the *S* = 9/2 state (Supplementary Table 4), with the two states differing only in the orientation (spin-up or -down) of the unpaired electrons in the 5*f* orbitals. The *S* = 11/2 and 13/2 states are 28.3 and 39.0 kcal mol^−1^ higher in energy, respectively, than the *S* = 9/2 state with RASSCF and include excitations out of the δ-bonding orbitals. Note that the number of determinants required for spin states lower than *S* = 7/2 rendered such calculations computationally intractable with this active space; therefore, a smaller active space was employed in which the δ-bonding orbitals with the highest occupation numbers (and their corresponding anti-bonding orbitals) were removed from the active space. In that scenario, CASPT2 calculations could be performed with an active space of (9*e*,17*o*) for spin states ranging from *S* = 1/2 to 9/2. Ultimately, these states are separated by at most 0.7 kcal mol^−1^ at the CASPT2 level of theory, and therefore are within the error of the method (Supplementary Table 10). These states, and other low-lying excited states that we have not explored, likely couple via spin–orbit interactions, but nevertheless the accuracy of CASPT2 is not sufficient to conclusively determine the ordering of the lowest states in such a complex multimetallic array. Since the results for **3** and **4** are expected to be similar, only **4‴** was studied with RASSCF and CASPT2. All RASSCF and CASPT2 calculations were performed with Molcas v7.

**Fig. 7:**
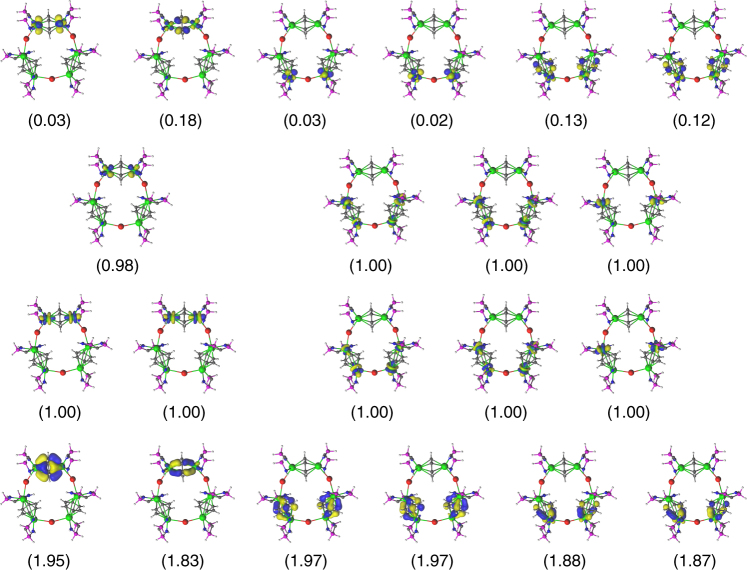
Active natural orbitals from a RASSCF calculation on **4‴**. Electron occupation numbers are given in parentheses. Key: uranium, green; iodide, red; phosphorus, purple; nitrogen, dark blue; carbon, grey; hydrogen, white

From the orbital picture that emerges in the RASSCF calculations, a similar bonding scheme is present for each U–arene–U group in **4‴**. Each of the U–C_6_H_6_–U groups contains two sets of δ-bonds (Fig. 7) and a set of singly occupied orbitals that are linear combinations of uranium 5*f* orbitals. The δ-bonds in these systems can be thought of as δ-backbonding where the uranium 5*f* orbitals donate into the π* orbitals on the arene^56–63^. Each U–C_6_H_6_–U group contains one δ-bond natural orbital with an occupation number of 1.95–1.97 while the other has a lower occupation number of 1.83–1.88. In Fig. 7, note that the δ-bond in the U–C_6_H_6_–U group at the top is symmetric, whose structural data suggest that it involves two uranium(IV) ions, while the δ-bonds in the two symmetry equivalent U–C_6_H_6_–U groups are asymmetric, which again is consistent with the overall presence of U^III^–arene–U^IV^ units.

However, the most important difference between **2** and **4** is the number of unpaired electrons per uranium ion. In **2**, four unpaired electrons remain localised on the two uranium(III) centres; note that uranium(III) ions when δ-bonded to arenes exhibit only two clear-cut, localised 5*f* electrons since the other 5*f* electron per uranium is involved to some extent in δ-bonding. The U–C_6_H_6_–U group shown at the top of **4‴** in Fig. 7 is symmetrically distinct from the other two U–C_6_H_6_–U groups and has three unpaired electrons distributed among the two uranium ions. As a result, the formal oxidation state of each uranium in **4‴** is higher than in **2**, consistent with the top two uranium ions being (IV). The remaining two U–C_6_H_6_–U groups contain six unpaired electrons distributed among the four uranium centres, reflecting the presence, formally, of three uranium(III) and one uranium(IV) ion in that grouping. Furthermore, the average Mulliken charge on the uranium ions is slightly higher in **4‴** (+1.49) than in **2‴** (+1.30) reflecting the presence of uranium(III) and (IV) ions in the former but only uranium(III) in the latter.

**Fig. 8:**
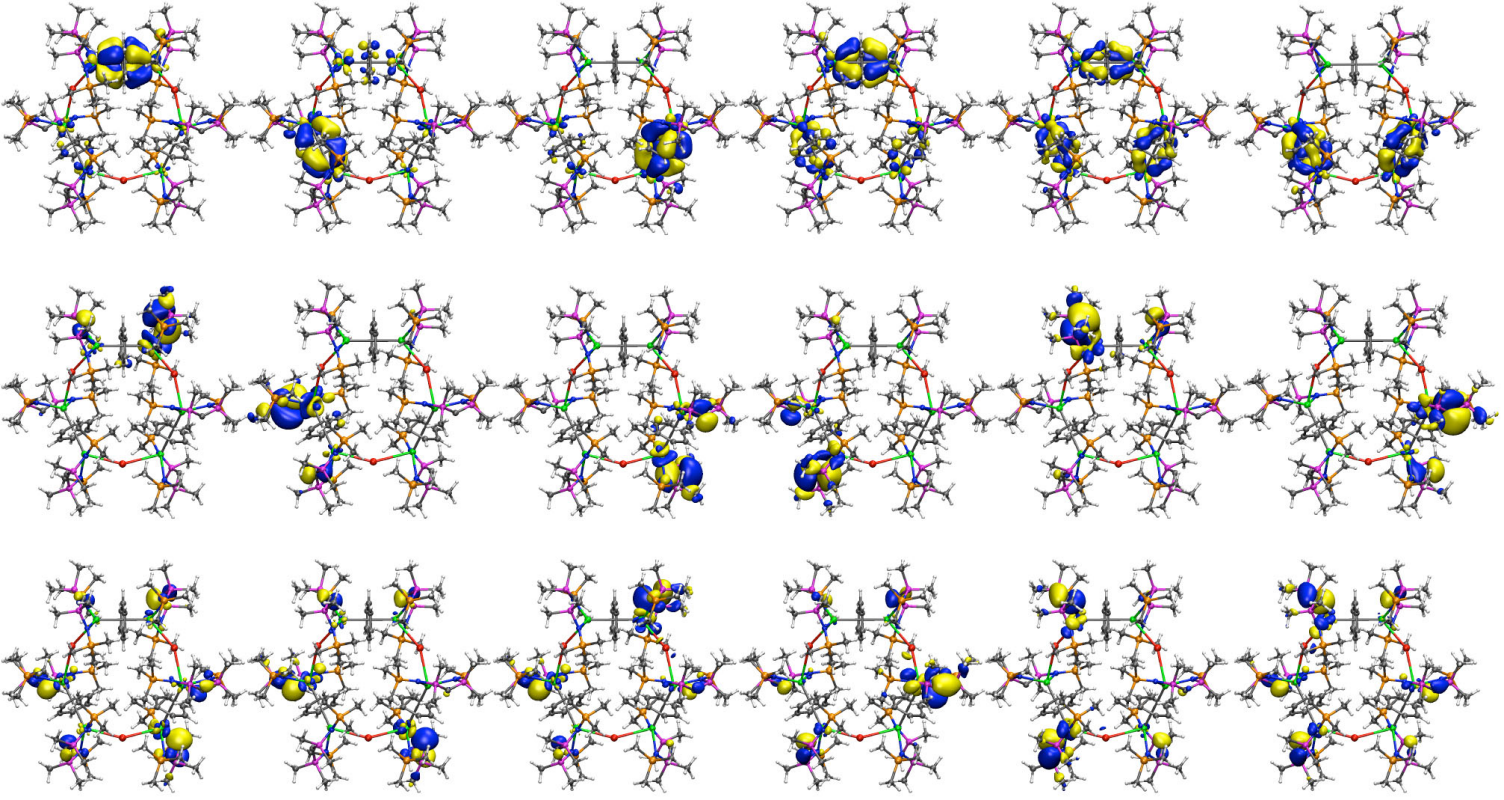
Computed DFT molecular orbitals for **4′**. Top row: the six uranium–arene δ-bonding interactions. Middle and bottoms rows: the 12 U = C *π*-double bond interactions. Key: uranium, green; iodide, red; phosphorus, purple; nitrogen, dark blue; carbon, grey; hydrogen, white

The RASSCF calculations naturally focus on the role of the arene groups, since those orbitals lie closest to the valence region, and thus contributions from the BIPM ligand were not included in the active space. However, turning to the uranium–BIPM interactions, the orbitals involved in the U–C_BIPM_ interaction are the next set of orbitals below the active space, but are energetically well separated lying lower in energy by ~30 kcal mol^−1^. To interrogate this group of interactions in more detail, we performed DFT (PBE/TZ2P and PBE/def-TZVP) calculations on two larger models, [{U(BIPM^L′^)}_6_(*μ*-I)_3_(*μ*-*η*^6^:*η*^6^-C_7_H_8_)_3_] (**3**′, BIPM^L′^ = C(PMe_2_NSiMe_3_)_2_) and [{U(BIPM^L′^)}_6_(*μ*-I)_3_(*μ*-*η*^6^:*η*^6^-C_6_H_6_)_3_] (**4**′, BIPM^L′^ = C(PMe_2_NSiMe_3_)_2_) to explore the nature of the U–C_BIPM_ interaction. At the DFT level, only the high spin *S* = 9/2 state was computed for **3**′ and **4**′ since lower spin states are spin-contaminated. The 12 orbitals with contributions primarily from the C_BIPM_ 2*p* orbitals, but also with contributions from the uranium centres, are shown in Fig. 8. The bonding between the uranium ions and the bridging arenes imposes an orientation upon the uranium ions, and for this reason the interaction between uranium and C_BIPM_ centres does not exhibit idealised *π*-orbitals; however, there are two doubly-occupied orbitals per uranium indicating double bond interactions. Supporting this notion, Nalewajski–Mrozek bond orders for U = C_BIPM_ give values ranging from 1.15 to 1.16 for **3**′ and 1.16 to 1.18 for **4**′; U = C_BIPM_ bond orders tend to be 1.23–1.54 with U–C_BIPMH_ bond orders being ~0.6 (ref. ^50^). Additionally, the average charges on the uranium centres are computed with the multiple derived charge analysis (MDC-q) to be +1.19 for **3**′ and +1.18 for **4′**. The molecular orbitals in **4′** that principally represent the U = C double bond interactions exhibit variable levels of intrusion from other orbital coefficients due to their delocalised nature, but despite this from the more localised and thus clear-cut U = C molecular orbital combinations a clear trend emerges; the uranium(III)-, uranium(III/IV)-, and uranium(IV)-C_BIPM_ bonds show uranium and carbon contributions of 14:57%, 18:40%, and 22:20%, respectively where the total uranium contribution is clearly increasing with increasing oxidation state. Unfortunately, due to the large number of basis functions Natural Bond Orbital analysis was not performed. For similar reasons, although QTAIM data can be computed these metrics should be appraised in a qualitatively global sense rather than in fine detail. Nevertheless, *ρ*, ∇^2^*ρ*, *H*, and *ε* (ellipticity) values of 0.058/0.056, 0.158/0.154, 0.09/0.08, and 0.20/0.19 for uranium(III)/uranium(IV)-carbon 3,−1-critical points, respectively, clearly indicate the presence of U = C multiple-bond interactions since for the latter parameter cylindrical single or triple bonds have *ε-*values of ~0 but asymmetric double bonds tend to exhibit *ε-*values of 0.1–0.6 (ref. ^71^).

## Discussion

The BIPM^TMS^ ligand clearly stabilises high oxidation states of uranium, as evidenced by prior reports of derivatives with uranium in oxidation states of IV, V, and VI^47,50^. For **2**, the putative uranium(III) intermediate is destabilised by the BIPM^TMS^ ligand^43^, which abstracts a proton from solvent to relax to the methanide form of BIPM^TMS^. This notion is supported by electrochemical reduction of **1** that results in disproportionation, which is indirect evidence for an unstable uranium(III)-methanediide. Further, when THF solutions of the uranium(III)-methanide [U(BIPM^TMS^H)(I)_2_(THF)] are reacted with benzyl potassium, the characteristic dark blue-purple colour of uranium(III) is observed for short periods, putatively assigned as the uranium(III)-methanediide [U(BIPM^TMS^)(I)(THF)_2_], but disproportionation and deposition of uranium(0) with a colour change to brown, typical of uranium(IV), is observed. These observations demonstrate how unfavourable the combination of uranium(III) and the BIPM^TMS^ methanediide ligand are, which relates to the general mismatch of an electron-rich ligand to a low oxidation state metal. Thus, when the methanediide cannot convert to a methanide it is more difficult to reduce the uranium ions, which accounts for the fact that uranium(IV) ions are present in the **3** and **4**, even with a large excess of KC_8_, whereas only uranium(III) ions are found in **2**.

The formation of the hexauranium rings in this report is notable because the assembly of an arene complex nicely provides a mechanism by which to cushion the, relatively speaking for uranium, electron-rich nature of uranium(III) combined with electron-rich methanediide ligands that even in toluene might be expected to result in deprotonation of the acidic methyl of toluene resulting in a methanide like **2** (ref. ^43^). Thus, the arene δ-bonding interactions can be viewed as a reservoir that can accommodate charge from uranium, thereby avoiding destabilisation with respect to disproportionation and giving relatively stable metal-ligand multiple bonds. Metal-arene functional groups are usually the focus of a molecule, whereas here they are performing a stabilising ancillary role to support another metal-ligand functional unit. This stabilisation strategy could constitute a general synthetic strategy by which to stabilise reactive metal-ligand linkages. This could be either in the manner described here where the arene is an acceptor ligand, or even in an inverse scenario with electron-poor co-ligands where arenes act as donor ligands. This of course depends on the nature of the arene, metal, co-ligands, and oxidation state, and thus electron richness, of the metal, but as a purposefully deployed synthetic strategy could enable electronic stabilisation of otherwise inaccessible reactive linkages.

That the toluene ligands in **3** can be exchanged with benzene to return **4** at all without total decomposition of the ring motifs is remarkable, especially given the replacement of toluene by benzene can be observed stepwise. The toluene–benzene exchange is consistent with the presence of uranium(III) ions^58^, since benzene is a better electron acceptor ligand than toluene.

The crystallographic data for **3** reveal distinct, rather than averaged, U = C distances that are consistent with the presence of discrete uranium(III) and uranium(IV) ions. Complex **3** can thus be classified as a Robin Day Class I system, which is possibly surprising given the linking of uranium ions via δ-bonded arenes. The presence of BIPM^TMS^ disordered over two sites at the bottom of the ring also suggests clear-cut, rather than delocalised, uranium(III) and uranium(IV) ions. The halide distances also fall into long, short, and intermediate distances. The U–I distance associated with the disordered U1–BIPM fragment cannot be considered reliable in detail, but when the BIPM methanediide binds closely to uranium the iodide binds at a longer distance because the methanediide is the strongest donor. The U–arene interactions do not seem to adjust significantly, consistent with their buffering, reservoir role.

The electronic absorption spectrum of **3** contains signature absorptions of uranium(III)^43^. Further, there is no evidence for IVCT in **3** and for **2** where there should not be any IVCT. The EPR spectrum of **3** is only found at low (<30 K) temperature, which is characteristic of f-electrons that can rapidly relax due to large orbital angular momentum^66^. Since uranium(IV) is typically EPR-silent due to its non-Kramers nature, the observation of an EPR spectrum at all is consistent with the presence of uranium(III) Kramers ions. The magnetism also supports the presence of formal uranium(III) ions due to the observed low temperature limiting value of the magnetic moment of **3**, and the *M* vs *H* magnetisation data which readily saturate. XANES data, whether the white line is determined from the first or second derivative of the data, consistently place **3** in between uranium(III) and uranium(IV) standards suggesting the presence of both ions^67–69^. Mixed valence actinide(III/IV) complexes remain rare^72–76^, and we are not aware of any XANES studies of such species^68^, but the presence of Robin Day Class I or III systems has been inferred mostly by crystallographic data^72–76^. A single absorption feature for **3** and **4** does not rule out a Class I assignment for these complexes because the observed feature is a linear combination of the two oxidation state spectra that would have to be significantly different to deconvolute. This is not the case for organo-uranium(III) and -uranium(IV) since their energy separations are relatively small, and so the resulting combined spectrum has the appearance of the end-member states. Indeed, single feature XANES spectra have been observed for Class I Ag(I)/Ag(II) complexes^77^.

As might be expected, RASSCF and CASSCF/CASPT2 calculations on **4**‴ indicate that there are several spin states that lie very close in energy to one another. Nevertheless, the lowest lying states all have nine unpaired electrons (with different combinations of spin-up and -down), which is consistent with the magnetisation studies. An important feature of the calculations is that they identify U^III^–arene–U^IV^ units rather than a delocalised picture of +3.5 uranium ions. On the other hand, the bonding in the DFT calculations are inherently fully delocalised and the molecular orbitals for the unpaired electrons and δ−bonds are equivalent for all three U–arene–U groups, suggesting that RASSCF calculations are important for accurately describing the bonding in the U–arene–U groups.

The calculations clearly identify a group of molecular orbitals under the uranium–arene interactions that represent the U = C multiple-bond interactions. As highlighted recently^78^, there has been some controversy over the nature of actinide-methanediide bonding with these geminal dianions since it has been suggested that the M^+^-C^−^ resonance form dominates^79^. However, an extensive body of work consistently returns uranium–carbon multiple bonds through a variety of calculated measures (DFT, NBO, QTAIM)^36,38–40,42,44–48,50–54^ and also by experimentally (NMR) verified computations for thorium congeners^80^. DFT and NBO frequently find two orbitals with uranium and carbon character for each U = C bond, and computed charges and bond orders support the presence of multiple bonds^47,50^. Further, QTAIM often returns asymmetric bond ellipticities at the U = C bond critical points (BCPs)—note these BCPs would not be present at all if the electrons in those bonds were localised at the carbon—which can only be the case if a double bond is present because single and triple bonds present symmetric bond ellipticities; if a U^+^–C^−^ dipolar interaction were the dominant resonance form this would equate to a σ-bond with symmetric bond ellipticity since a localised carbon lone pair would not contribute to the BCP^71^. To put the U = C ellipticity data in this work in a wider context, the ellipticity value of a C–C σ-bond is zero, the C–C bonds of benzene are 0.23, and the C–C bond in ethylene is 0.45 (ref. ^71^). Similar findings have been reported for thorium^78,80^, and although the phosphorano groups of BIPM^TMS^ attenuate the U = C bonds it is clear from computed data, in particular the non-zero bond critical point ellipticity data, and inferred from paramagnetic shielding data in NMR computations on analogous thorium complexes^80^, that two-fold polarised-covalent U = C multiple bonding interactions are present in **3** and **4**. The fact that polarised-covalent U = C multiple bonds are identified in calculations on **3′** and **4′** is thus consistent and notable, and computed U = C bond orders of ca 1.16 and 1.17, respectively, are consistent with a two-fold polarised U = C bonding interactions where each σ- and *π*-component are less than one because they are polarised. The computed molecular orbitals that represent the principal U = C bonding interactions are by definition delocalised and so their percentage breakdowns are not meaningful in an absolute sense. However, the derived uranium and carbon percentage contributions build a consistent, qualitative trend of increasing uranium contribution on moving from uranium(III) to uranium(IV) with the disordered uranium(III/IV) averaged mixture sitting in the middle. This nicely fits the established trend of increasing uranium contribution to U = C bonds on increasing oxidation state (av. 17.5, 25.8, and 28.1% for uranium(IV/V/VI), respectively, by NBO)^47,50^ from which we estimate, since NBO calculations are not feasible, that the uranium(III) contributions correspond to ~13% of the U = C bond.

Probing complexes like **3** and **4** is a formidable task, and thus arriving at a clear-cut assessment of their electronic structure is inherently challenging. However, the arene is acting as an electronic buffer, and there are clearly polarised-covalent U = C double bonds. Some data are consistent with the presence of uranium(III) ions: (i) there are distinct, not averaged, U = C distances with disordered BIPM^TMS^ representing separate uranium sites in the crystal structures; (ii) the arene exchange reactions are consistent with uranium(III) arene reactivity; (iii) optical data show characteristic uranium(III) absorptions and no IVCT; (iv) *M* vs *H* magnetic data saturate; (v) EPR data show the presence of Kramers ions; (vi) multi-reference calculations clearly show discrete U^III^–arene–U^III^ and U^III^–arene–U^IV^ units. However, some data do not permit us to fully rule out a delocalised mixed-valence system: (i) the U–I distances follow an opposite trend to the U = C bonds, but this can be related to relative ligand donor strengths; (ii) magnetic and EPR data confirm the presence of Kramers ions, but this itself does not rule out delocalisation; (iii) XANES data are a composite, as seen in other Class I systems, that shows a mixture of uranium(III) and uranium(IV) but not whether they are localised or delocalised; (iv) DFT presents a more delocalised picture, but this is inherent to DFT. It is certainly the case that not all the uranium ions in **3** and **4** are uranium(IV), and at the very least the U = C bonds involve uranium ions that are lower oxidation state than found previously. So, most of the data suggest the presence of formal uranium(III) ions supported by δ-bonding to the arenes, but at this point we cannot completely rule-out the possibility of electron delocalisation in this Kramers system.

## Methods

### General

Experiments were carried out under a dry, oxygen-free dinitrogen atmosphere using Schlenk-line and glove-box techniques. All solvents and reagents were rigorously dried and deoxygenated before use. Compounds were variously characterised by elemental analyses, electrochemistry, NMR, FTIR, EPR, XANES, and UV/Vis/NIR electronic absorption spectroscopies, single crystal X-ray diffraction studies, Evans methods and SQUID magnetometry, and DFT, QTAIM, CASSCF, CASPT2, and RASSCF computational methods.

### Preparation of [{U(BIPM^TMS^)(*μ*-I)_0.5_(*μ*-*η*^6^:*η*^6^-C_7_H_8_)_0.5_}_6_] (**3**)

Toluene (20 ml) was added to a pre-cooled (–78 °C) mixture of **1** (1.22 g, 0.5 mmol) and KC_8_ (0.27 g, 2 mmol). The reaction mixture was slowly allowed to warm to room temperature with stirring over 24 h. The mixture was filtered, reduced in volume to ca. 5 ml, and stored at ambient temperature overnight to afford **3** as dark purple crystals. Yield 0.12 g, 12%. Anal Calcd for C_207_H_252_I_3_N_12_P_12_Si_12_U_6_._4_C_7_H_8_: C, 48.01; H, 4.88; N, 2.95. Found: C, 48.03; H, 4.94; N, 2.66. ^1^H NMR (d6-benzene, 298 K): δ –1.96 (s, br, 12 H, Ph-CH), –1.35 (s, 9 H, toluene Ph-CH), –0.86 (s, br, 54 H, Si(CH3)3), –0.37 (s, 6 H, toluene Ph-CH), 0.44 (s, 9 H, toluene CH_3_), 4.39 (s, 12 H, Ph-CH), 4.85 (s, 24 H, Ph-CH), 5.39 (s, 24 H, Ph-CH), 5.75 (s, 12 H, Ph-CH), 10.38 (s, 6 H, Ph-CH), 10.83 (s, 12 H, Ph-CH), 14.11 (s, 12 H, Ph-CH), 14.53 (s, br, 54 H, Si(CH_3_)_3_), 14.96 (s, 12 H, Ph-CH). FTIR v/cm^−1^ (Nujol): 1650 (w), 1572 (w), 1243 (m), 1103 (m), 1033 (s), 830 (m), 763 (m), 691 (m), 643 (m), 604 (m), 555 (m), 502 (w).

### Preparation of [{U(BIPM^TMS^)(*μ*-I)_0.5_(*μ*-*η*^6^:*η*^6^-C_6_H_6_)_0.5_}_6_] (**4**)

Benzene (20 ml) was added to a pre-cooled (4 °C) mixture of **1** (3.79 g, 1.66 mmol) and KC_8_ (0.90 g, 6.63 mmol). The reaction mixture was slowly allowed to warm to room temperature with stirring over 24 h. The mixture was filtered, reduced in volume to ca. 3 ml, and stored at 5 °C overnight to afford **4** as dark purple crystals. Yield 0.04 g, 1%. Anal Calcd for C_204_H_246_I_3_N_12_P_12_Si_12_U_6_: C, 45.50; H, 4.61; N, 3.12. Found: C, 45.47; H, 4.87; N, 2.77. ^1^H NMR (*d*_6_-benzene, 298 K): δ –1.89 (s, br, 24 H, Ph-C*H*), –1.24 (s, br, 6 H, Ph-C*H*), –0.98 (s, br, 54 H, Si(C*H*_3_)_3_), –0.25 (s, 18 H, benzene Ph-C*H*), 3.49 (s, 12 H, Ph-C*H*), 5.09 (s, 24 H, Ph-C*H*), 5.92 (s, 24 H, Ph-C*H*), 6.30 (s, 12 H, Ph-C*H*), 10.53 (s, 6 H, Ph-C*H*), 11.28 (s, 12 H, Ph-C*H*), 15.36 (s, br, 54 H, Si(C*H*_3_)_3_). FTIR v/cm^−1^ (Nujol): 1590 (w), 1534 (w), 1304 (m), 1153 (m), 918 (m), 770 (m), 692 (m), 675 (m), 658 (m), 642 (m), 603 (m), 554 (m), 505 (w).

### Data availability

The X-ray crystallographic coordinates for structures reported in this article have been deposited at the Cambridge Crystallographic Data Centre (CCDC), under deposition number CCDC 1581465 (**3**) and 1581466 (**4**), These data can be obtained free of charge from The Cambridge Crystallographic Data Centre via www.ccdc.cam.ac.uk/data_request/cif. All other data can be obtained from the authors on request.

## Electronic supplementary material

Supplementary Information

## References

[1] Dam HH, Reinhoudt DN, Verboom W (2007). Multicoordinate ligands for actinide/lanthanide separations. Chem. Soc. Rev..

[2] Kozimor SA (2009). Trends in covalency for d- and f-element metallocene dichlorides identified using chlorine K-edge X-ray absorption spectroscopy and time-dependent density functional theory. J. Am. Chem. Soc..

[3] Seaman LA (2012). Probing the 5f orbital contribution to the bonding in a U(V) ketimide complex. J. Am. Chem. Soc..

[4] Minasian SG (2012). Determining relative f and d orbital contributions to M-Cl covalency in MCl_6_^2−^ (M=Ti, Zr, Hf, U) and UOCl_5_^−^ using Cl K-edge X-ray absorption spectroscopy and time-dependent density functional theory. J. Am. Chem. Soc..

[5] Spencer LP (2013). Tetrahalide complexes of the [U(NR)^2]2+^ ion: synthesis, theory, and chlorine K-edge X-ray absorption spectroscopy. J. Am. Chem. Soc..

[6] Lukens WW (2013). Quantifying the σ and π interactions between U(V) f orbitals and halide, alkyl, alkoxide, amide, and ketimide ligands. J. Am. Chem. Soc..

[7] Vitova T (2017). The role of the 5f valence orbitals of early actinides in chemical bonding. Nat. Commun..

[8] Formanuik A (2017). Actinide covalency measured by pulsed electron paramagnetic resonance spectroscopy. Nat. Chem..

[9] Hayton TW (2010). Metal-ligand multiple bonding in uranium: structure and reactivity. Dalton Trans..

[10] Hayton TW (2013). Recent developments in actinide-ligand multiple bonding. Chem. Commun..

[11] Jones MB, Gaunt AJ (2013). Recent developments in synthesis and structural chemistry of nonaqueous actinide complexes. Chem. Rev..

[12] La Pierre, H. S. & Meyer, K. in *Progress in Inorganic Chemistry*, Vol. **58** (ed. Karlin, K. D.) 303–416 (John Wiley & Sons, Inc., Hoboken, New Jersey, 2014).

[13] Liddle ST (2015). The renaissance of non-aqueous uranium chemistry. Angew. Chem. Int. Ed..

[14] Chatelain L, Scopelliti R, Mazzanti M (2016). Synthesis and structure of nitride-nridged uranium(III) complexes. J. Am. Chem. Soc..

[15] Nakai H, Hu X, Zakharov LN, Rheingold AL, Meyer K (2004). Synthesis and characterization of *N*-heterocyclic carbene complexes of uranium(III). Inorg. Chem..

[16] Seed JA (2017). Rare earth- and uranium-mesoionic carbenes: a new class of f-block carbene complex derived from an N-heterocyclic carbene. Angew. Chem. Int. Ed..

[17] Brennan JG, Andersen RA, Robbins JL (1986). Preparation of the first molecular carbon monoxide complex of uranium, (Me_3_SiC_5_H_4_)_3_UCO. J. Am. Chem. Soc..

[18] Parry J, Carmona E, Coles S, Hursthouse M (1995). Synthesis and single crystal X-ray diffraction study on the first isolable carbonyl complex of an actinide, (C_5_Me_4_H)_3_UCO. J. Am. Chem. Soc..

[19] Evans WJ, Kozimor SA, Nyce GW, Ziller JW (2003). Comparative reactivity of sterically crowded nf^3^ (C_5_Me_5_)_3_Nd and (C_5_Me_5_)_3_U complexes with CO: formation of a nonclassical carbonium ion versus an f element metal carbonyl complex. J. Am. Chem. Soc..

[20] Evans WJ, Kozimor SA, Ziller JW (2003). A monometallic f element complex of dinitrogen: (C_5_Me_5_)_3_U(*η*-N_2_). J. Am. Chem. Soc..

[21] Siladke NA (2012). Synthesis, structure, and magnetism of an f element nitrosyl complex, (C_5_Me_4_H)_3_UNO. J. Am. Chem. Soc..

[22] Cramer RE, Maynard RB, Paw JC, Gilje JW (1981). A uranium-carbon multiple bond. Crystal and molecular structure of (*η*^5^-C_5_H_5_)_3_UCHP(CH_3_)_2_(C_6_H_5_). J. Am. Chem. Soc..

[23] Cramer RE, Higa KT, Pruskin SL, Gilje JW (1983). Uranium-carbon multiple-bond chemistry. 2. Coupling of bridging and terminal carbonyls in the formation of an iron *η*^1^:*η*^3^-allyl complex. J. Am. Chem. Soc..

[24] Cramer RE, Panchanatheswaran K, Gilje JW (1984). Isocyanide insertion into a uranium-carbon double bond. Angew. Chem., Int. Ed..

[25] Cramer RE, Panchanatheswaran K, Gilje JW (1984). Uranium-carbon multiple-bond chemistry. 3. Insertion of acetonitrile and the formation of a uranium-nitrogen multiple bond. J. Am. Chem. Soc..

[26] Cramer RE, Higa KT, Gilje JW (1984). Uranium-carbon multiple-bond chemistry. 4. Addition of coordinated carbon monoxide across a uranium-carbon multiple bond. J. Am. Chem. Soc..

[27] Lyon JT, Andrews L (2006). Formation and characterization of the uranium methylidene complexes CH_2_=UHX (X=F, Cl, and Br). Inorg. Chem..

[28] Lyon JT (2007). Infrared spectrum and bonding in uranium methylidene dihydride, CH_2_=UH_2_. Inorg. Chem..

[29] Roos BO, Lindh R, Cho HG, Andrews L (2007). Agostic Interaction in the methylidene metal dihydride complexes H_2_MCH_2_ (M=Y, Zr, Nb, Mo, Ru, Th, or U). J. Phys. Chem. A.

[30] Lyon JT, Andrews L, Hu HS, Li J (2008). Infrared spectra and electronic structures of agostic uranium methylidene molecules. Inorg. Chem..

[31] Cho HG, Lyon JT, Andrews L (2008). Reactions of actinide metal atoms with ethane: computation and observation of new Th and U ethylidene dihydride, metallacyclopropane dihydride, and vinyl metal trihydride complexes. J. Phys. Chem. A.

[32] Cho HG, Andrews L (2012). Infrared spectra of the *η*^2^-M(NC)-CH_3_, CH_3_-MNC, and CH_2_=M(H)NC complexes prepared by reactions of thorium and uranium atoms with acetonitrile. Organometallics.

[33] He MY, Xiong G, Toscano PJ, Burwell RL, Marks TJ (1985). Supported organoactinides. Surface chemistry and catalytic properties of alumina-bound cyclopentadienyl and pentamethylcyclopentadienyl thorium and uranium hydrocarbyls and hydrides. J. Am. Chem. Soc..

[34] Villiers C, Ephritikhine M (2001). Reactions of aliphatic ketones R_2_CO (R=Me, Et, *i*Pr, and *t*Bu) with the MCl_4_/Li(Hg) system (M=U or Ti): mechanistic analogies between McMurray, Wittig, and Clemmensen reactions. Chem. Eur. J..

[35] Yahia A, Castro L, Maron L (2010). A theoretical study of uranium(IV) bis-methyl complexes: towards the predictive formation of a transient uranium(IV) carbene complex. Chem. Eur. J..

[36] Cantat T (2009). The U=C double bond: synthesis and study of uranium nucleophilic carbene complexes. J. Am. Chem. Soc..

[37] Tourneux JC (2010). Easy access to uranium nucleophilic carbene complexes. Dalton Trans..

[38] Cooper OJ, McMaster J, Lewis W, Blake AJ, Liddle ST (2010). Synthesis and structure of [U{C(PPh_2_NMes)_2_}_2_] (Mes=2,4,6-Me_3_C_6_H_2_): a homoleptic uranium bis(carbene) complex with two formal U=C double bonds. Dalton Trans..

[39] Cooper OJ (2011). Uranium-carbon multiple bonding: facile access to the pentavalent uranium carbene [U{C(PPh_2_NSiMe_3_)_2_}(Cl)_2_(I)] and comparison of U^V^=C and U^IV^=C double bonds. Angew. Chem. Int. Ed..

[40] Tourneux JC (2011). Exploring the uranyl organometallic chemistry: from single to double uranium-carbon bonds. J. Am. Chem. Soc..

[41] Tourneux JC (2011). Uranium(IV) nucleophilic carbene complexes. Organometallics.

[42] Fortier S, Walensky JR, Wu G, Hayton TW (2011). Synthesis of a phosphorano-stabilized U(IV)-carbene via one-electron oxidation of a U(III)-ylide adduct. J. Am. Chem. Soc..

[43] Mills DP (2011). A delocalised arene-bridged diuranium single molecule magnet. Nat. Chem..

[44] Ma GB, Ferguson MJ, McDonald R, Cavell RG (2011). Actinide metals with multiple bonds to carbon: synthesis, characterization, and reactivity of U(IV) and Th(IV) bis(iminophosphorano)methandiide pincer carbene complexes. Inorg. Chem..

[45] Mills DP (2012). Synthesis of a uranium(VI)-carbene: reductive formation of uranyl(V)-methanides, oxidative preparation of a [R_2_C=U=O]^2+^ analogue of the [O=U=O]^2+^ uranyl ion (R=Ph_2_PNSiMe_3_), and comparison of the nature of U^IV^=C, U^V^=C and U^VI^=C double bonds. J. Am. Chem. Soc..

[46] Cooper OJ (2013). The nature of the U=C bond: pushing the stability of high oxidation state uranium carbenes to the limit. Chem. Eur. J..

[47] Ephritikhine M (2013). Uranium carbene compounds. C R Chim..

[48] Lu E (2014). Synthesis, characterization, and reactivity of a uranium(VI) carbene imido oxo complex. Angew. Chem. Int. Ed..

[49] Cooper OJ, Mills DP, Lewis W, Blake AJ, Liddle ST (2014). Reactivity of the uranium(IV) carbene complex [U(BIPM^TMS^)(Cl)(*μ*-Cl)_2_Li(THF)_2_] (BIPM^TMS^={C(PPh_2_NSiMe_3_)_2_}) towards carbonyl and heteroallene substrates: metallo-wittig, adduct formation, C-F bond activation, and [2+2]-cycloaddition reactions. Dalton Trans..

[50] Gregson M, Wooles AJ, Cooper OJ, Liddle ST (2015). Covalent uranium carbene chemistry. Comments Inorg. Chem..

[51] Gregson M (2016). Emergence of comparable covalency in isostructural cerium(IV) and uranium(IV)-carbon multiple bonds. Chem. Sci..

[52] Lu E, Tuna F, Lewis W, Kaltsoyannis N, Liddle ST (2016). Uranium metalla-allenes with carbene imido R_2_C=U^IV^=NR’ units (R=Ph_2_PNSiMe_3_; R’=CPh_3_): alkali metal-mediated push-pull effects with an amido auxiliary. Chem. Eur. J..

[53] Lu E (2016). Uranium-carbene-imido metalla-allenes: ancillary-ligand-controlled *Cis*-/*trans*-isomerisation and assessment of *trans*-influence in the R_2_C=U^IV^=NR’ unit (R=Ph_2_PNSiMe_3_; R’=CPh_3_). Chem. Eur. J..

[54] Gregson M (2017). The inverse-trans-influence in tetravalent lanthanide and actinide bis(carbene) complexes. Nat. Commun..

[55] Liddle ST, Mills DP, Wooles AJ (2011). Early metal bis(phosphorus-stabilised)carbene chemistry. Chem. Soc. Rev..

[56] Diaconescu PL, Arnold PL, Baker TA, Mindiola DJ, Cummins CC (2000). Arene-bridged diuranium complexes: inverted sandwiches supported by δ backbonding. J. Am. Chem. Soc..

[57] Vlaisavljevich B, Diaconescu PL, Lukens WL, Gagliardi L, Cummins CC (2013). Organometallics.

[58] Evans WJ, Kozimor SA, Ziller JW, Kaltsoyannis N (2004). Structure, reactivity, and density functional theory analysis of the six-electron reductant, [(C_5_Me_5_)_2_U]_2_(*μ*-*η*^6^:*η*^6^-C_6_H_6_), synthesized via a new mode of (C_5_Me_5_)_3_M reactivity. J. Am. Chem. Soc..

[59] Liddle ST (2015). Inverted sandwich arene complexes of uranium. Coord. Chem. Rev..

[60] MacDonald MR (2013). Identification of the+2 oxidation state for uranium in a crystalline molecular complex, [K(2.2.2-Cryptand)][(C_5_H_4_SiMe_3_)_3_U]. J. Am. Chem. Soc..

[61] La Pierre HS, Scheurer A, Heinemann FW, Hieringer W, Meyer K (2014). Synthesis and characterization of a uranium(II) monoarene complex supported by δ backbonding. Angew. Chem. Int. Ed..

[62] Patel D (2011). A formal high oxidation state inverse-sandwich diuranium complex: a new route to f-block-metal bonds. Angew. Chem. Int. Ed..

[63] Mougel V (2012). Siloxides as supporting ligands in uranium(III)-mediated small-molecule activation. Angew. Chem. Int. Ed..

[64] Evans WJ, Kozimor SA, Ziller JW (2005). Molecular octa-uranium rings with alternating nitride and azide bridges. Science.

[65] Kindra DR, Evans WJ (2014). Magnetic susceptibility of uranium complexes. Chem. Rev..

[66] Castro-Rodríguez I, Meyer K (2006). Small molecule activation at uranium coordination complexes: control of reactivity *via* molecular architecture. Chem. Commun..

[67] Kosog B, La Pierre HS, Denecke MA, Heinemann FW, Meyer K (2012). Oxidation state delineation via U L_III_-edge XANES in a series of isosstructural uranium coordination complexes. Inorg. Chem..

[68] Schelter EJ (2010). Comparative study of f-element electronic structure across a series of multimetallic actinide and lanthanoid-actinide complexes possessing redox-active bridging ligands. Inorg. Chem..

[69] Anderson NH (2015). Investigation of the electronic ground states for a reduced pyridine(diimine) uranium series: evidence for a ligand tetraanion stabilized by a uranium dimer. J. Am. Chem. Soc..

[70] Sauri V (2011). Multiconfigurational second-order perturbation theory restricted active space (RASPT2) method for electronic excited states: a benchmark study. J. Chem. Theory Comput..

[71] Bader RFW, Slee TS, Cremer D, Kraka E (1983). Descriptions of conjugation and hyperconjugation in terms of electron distributions. J. Am. Chem. Soc..

[72] Roussel P, Hitchcock PB, Tinker N, Scott P (1996). A mixed-valence uranium(III/IV) bimetallic; structure, magnetism and reactivity. Chem. Commun..

[73] Korobkov I, Gambarotta S, Yap GPA, Thompson L, Hay PJ (2001). Dinuclear trivalent and mixed-valence uranium [(-CH_2_-)_5_]_4_-calix[4]tetrapyrrole complexes with short intermetallic distances. Organometallics.

[74] Schelter EJ (2008). Mixed valency in a uranium multimetallic complex. Angew. Chem. Int. Ed..

[75] Larch CP, Cloke FGN, Hitchcock PB (2008). Activation and reduction of diethyl ether by low valent uranium: formation of the trimetallic, mixed valence uranium oxo species [U(Cp^RR’^)(*μ*-I)_2_]_3_(*μ*^3^-O) (Cp^RR’^=C_5_Me_5_, C_5_Me_4_H, C_5_H_4_SiMe_3_). Chem. Commun..

[76] Cary SK (2017). Incipient class II mixed valency in a plutonium solid-state structure. Nat. Chem..

[77] Mazej Z (2015). The first example of a mixed valence ternary compound of silver with random distribution of Ag(I) and Ag(II) cations. Dalton Trans..

[78] Rungthanaphatsophon P (2017). Formation of methane versus benzene in the reactions of (C_5_Me_5_)_2_Th(CH_3_)_2_ with [CH_3_PPh_3_]X (X=Cl, Br, I) yielding thorium-carbene or thorium-ylide complexes. Angew. Chem. Int. Ed..

[79] Levine DS, Tilley TD, Andersen RA (2017). Evidence for the existence of group 3 terminal methylidene complexes. Organometallics.

[80] Smiles DW, Wu G, Hrobárik P, Hayton TW (2017). Synthesis, thermochemistry, bonding, and ^13^C NMR chemical shift analysis of a phosphorano-stabilized carbene of thorium. Organometallics.

