# Supplementary material for: Uranium(III)-carbon multiple bonding supported by arene δ-bonding in mixed-valence hexauranium nanometre-scale rings
Source: Nat Commun. 2018 May 29;9:2097. doi: 10.1038/s41467-018-04560-7 (PMC5974406; doi:10.1038/s41467-018-04560-7)
Supplement: Supplementary file 1 — Supplementary Information [file 41467_2018_4560_MOESM1_ESM.pdf]

# Uranium(III)-Carbon Multiple Bonding Supported by Arene δ-Bonding in Mixed-Valence Hexauranium Nanometre-Scale Rings

Wooles et al.

**Supplementary Figures**

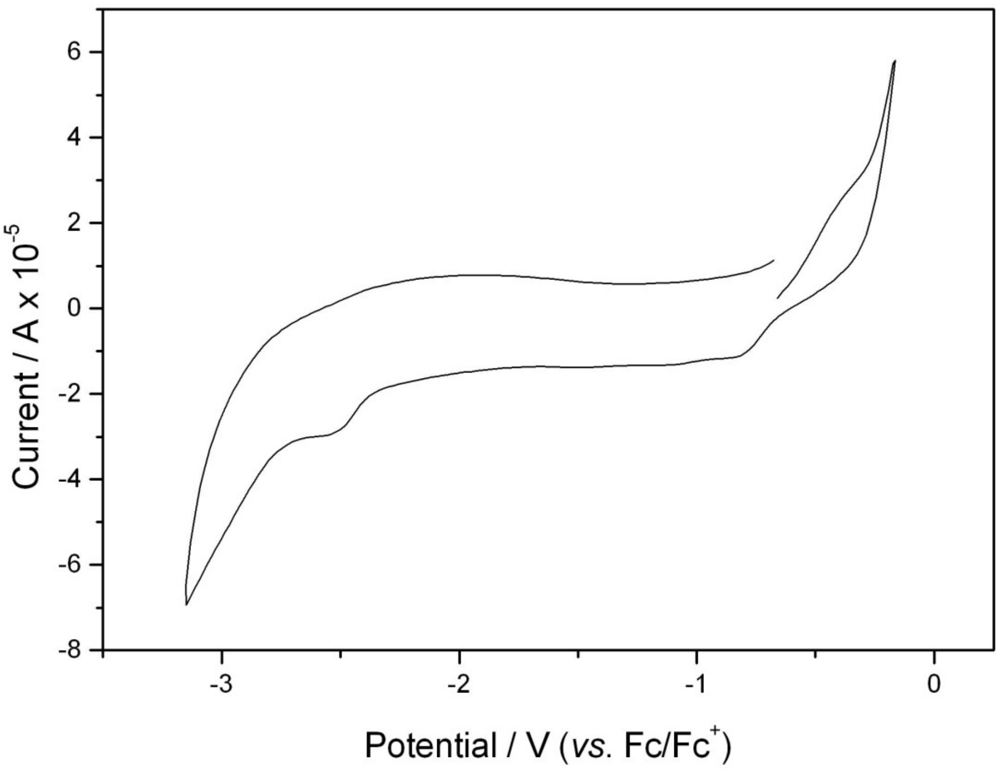


***Supplementary Figure 1.*** *Cyclic Voltammogram of* ***1*** *(1 mM) and [$NBu^n{}_4$][$BF_4$] (0.5 M) in THF. Scan rate 0.1 mV/s.*

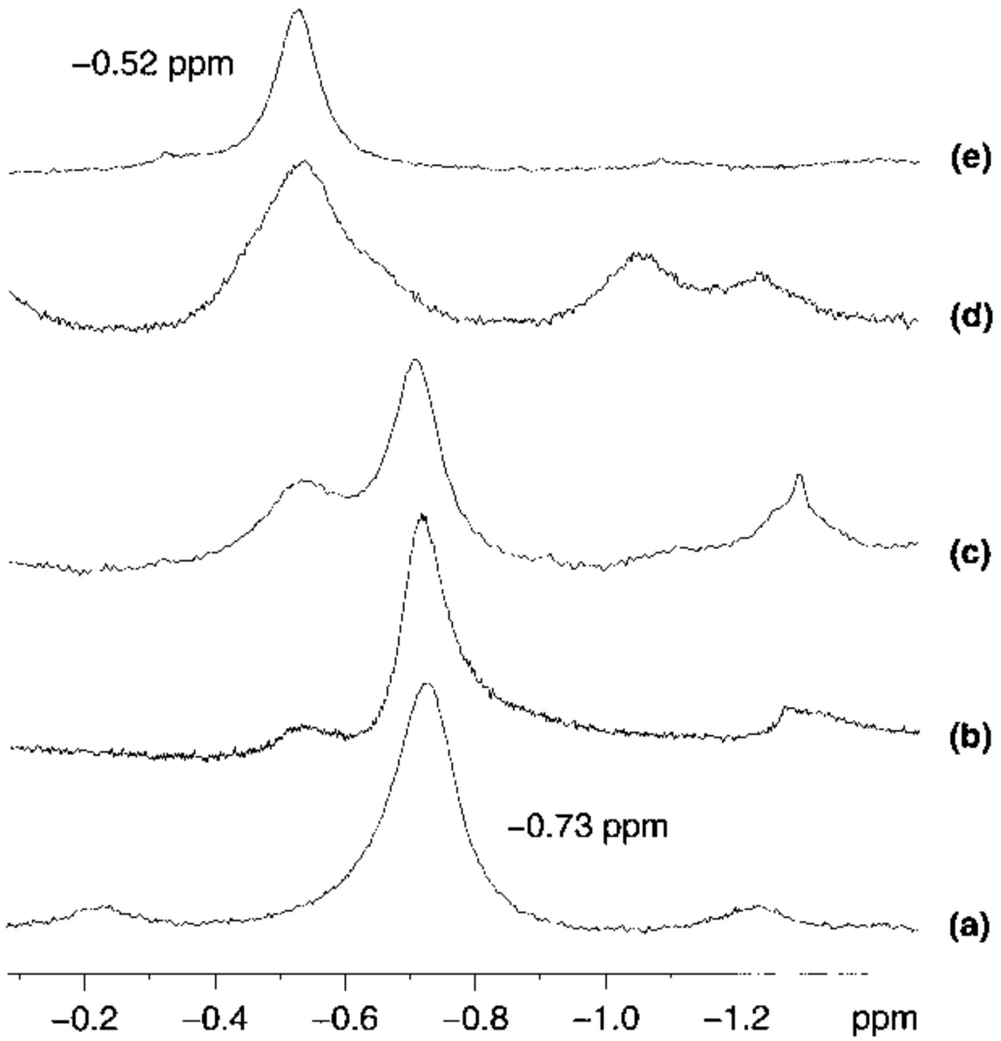


***Supplementary Figure 2.*** *Thermal conversion of complex **3** ($SiMe_3$ signal at –0.73 ppm) to **4**-$D_6$ ($SiMe_3$ signal at –0.52 ppm) at 50 °C measured by $^1H$ NMR spectroscopy; t = 0 hrs (a), 1 hr (b), 2 hrs (c), 4 hrs (d), 8hrs (e).*

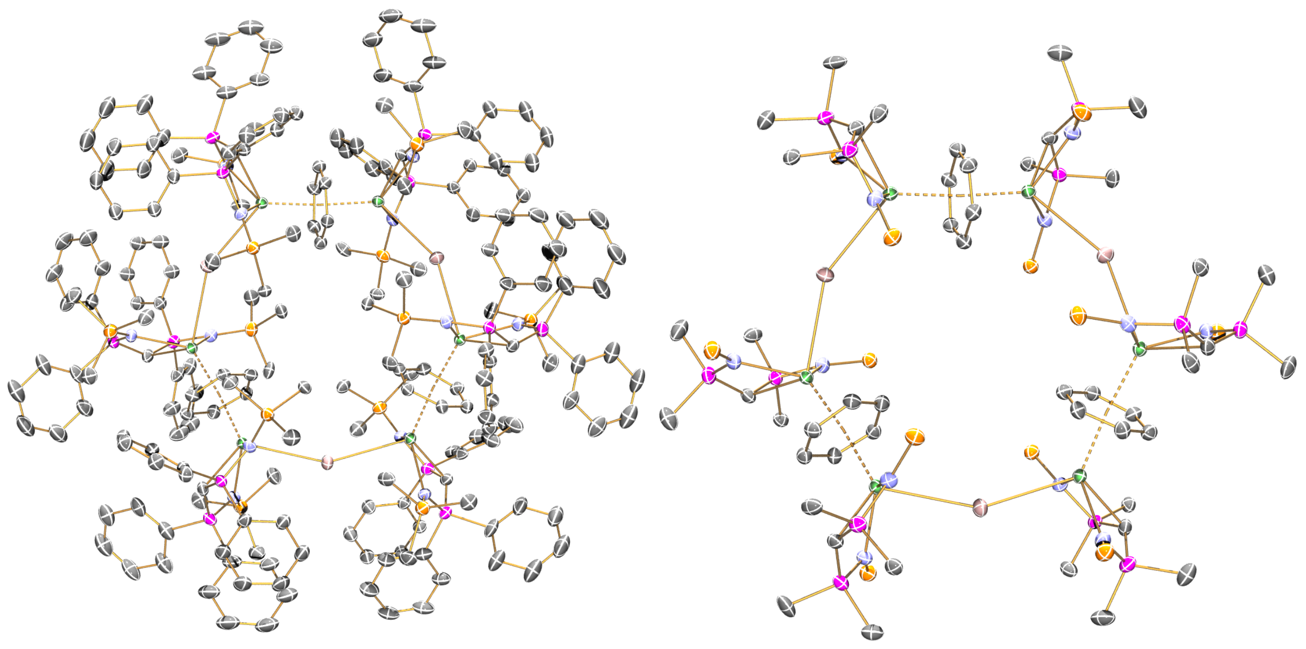

***Supplementary Figure 3.*** *Molecular structure of **4** at 90 K and displacement ellipsoids set to 40%. Full molecule on left and core on right. Key: uranium, green; phosphorus, magenta; silicon, orange; iodide, pink; nitrogen, blue; carbon, gray.*

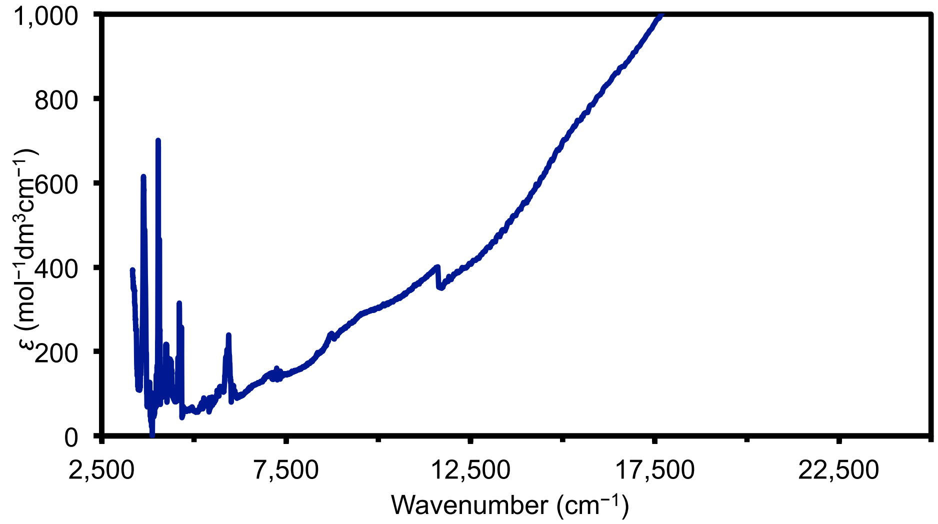


***Supplementary Figure 4.*** *UV/Vis/NIR spectrum of a 1 mM solution of complex* ***3*** *in toluene.*

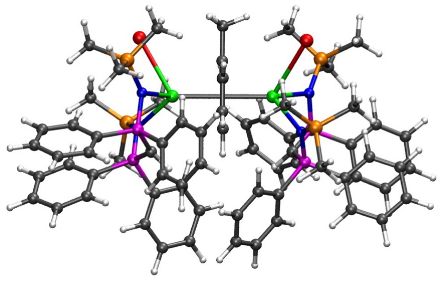
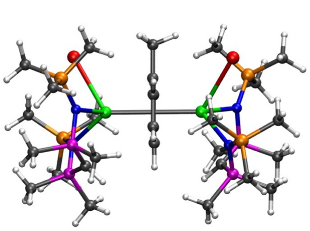
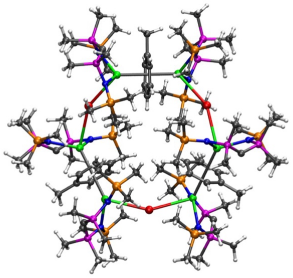

**2** **2'** **3'**

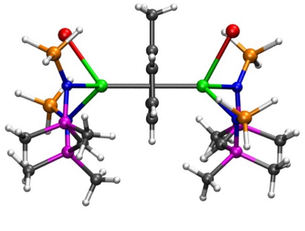
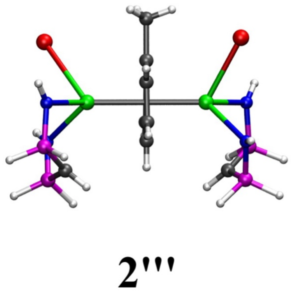
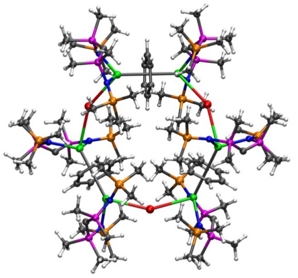
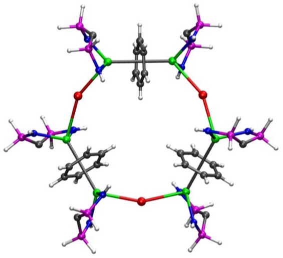

**2''** **2'''** **4'** **4'''**

***Supplementary Figure 5.*** *Ball and stick images of the computational models used in this study and their labels. For* ***2****: (BIPM$^{TMS}$ = C(PPh$_2$NSiMe$_3$)$_2$; For* ***2', 3'****, and* ***4'****: BIPM$^{L'}$ = C(PMe$_2$NSiMe$_3$)$_2$; for* ***2''****: BIPM$^{L''}$ = C(PMe$_2$NSiH$_3$)$_2$; and for* ***2'''*** *and* ***4'''****: BIPM$^{L'''}$ = C(PH$_2$NH)$_2$). Key: uranium, green; phosphorus, magenta; silicon, orange; iodide, pink; nitrogen, blue; carbon, gray.*

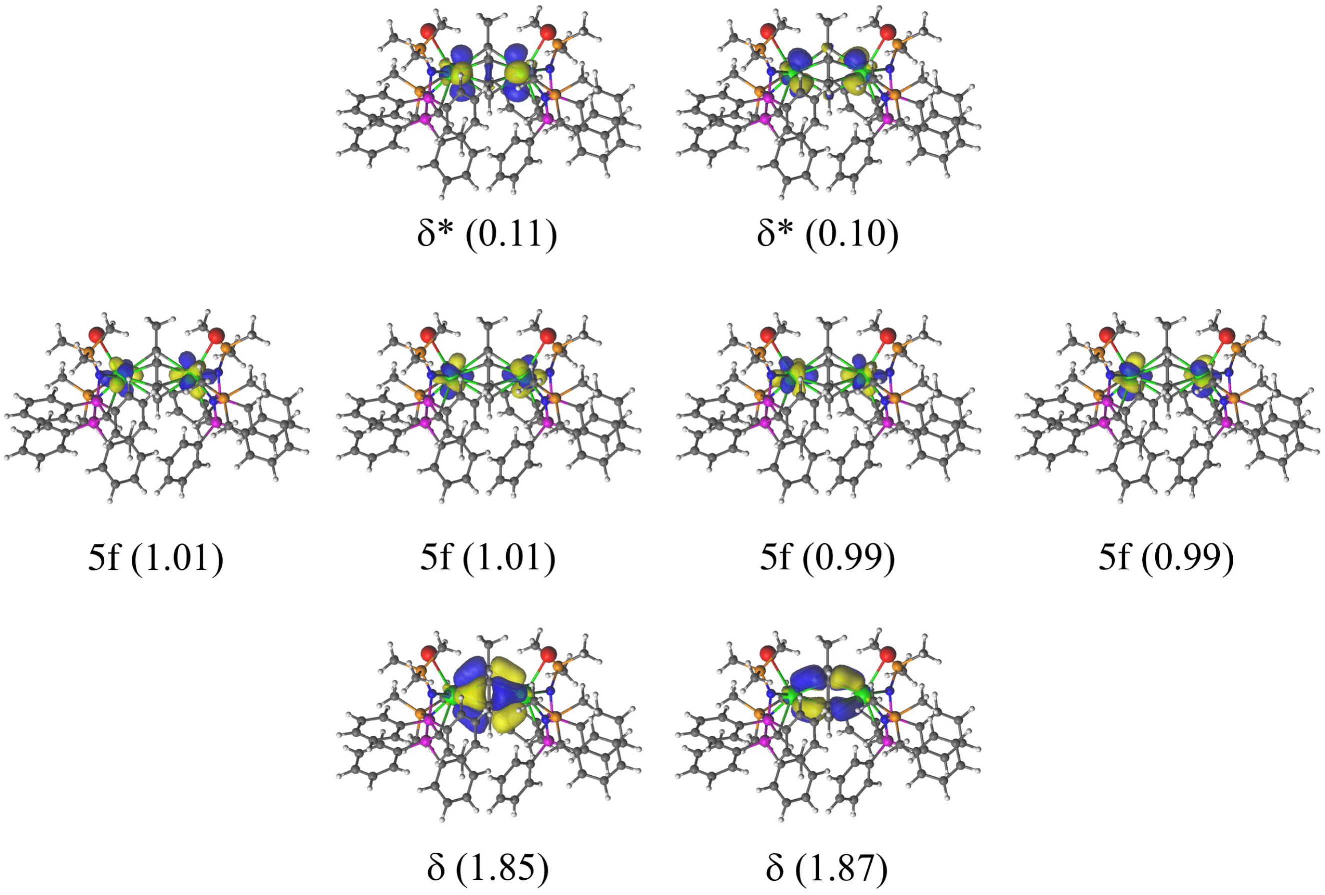


***Supplementary Figure 6.*** *The natural orbitals from the (8e,14o) active space of* ***2****. Occupation numbers are given in parentheses and the ANO-RCC basis set was used. Orbitals with occupation numbers less than 0.03 are not plotted. Key: uranium, green; phosphorus, magenta; silicon, orange; iodide, pink; nitrogen, blue; carbon, gray.*

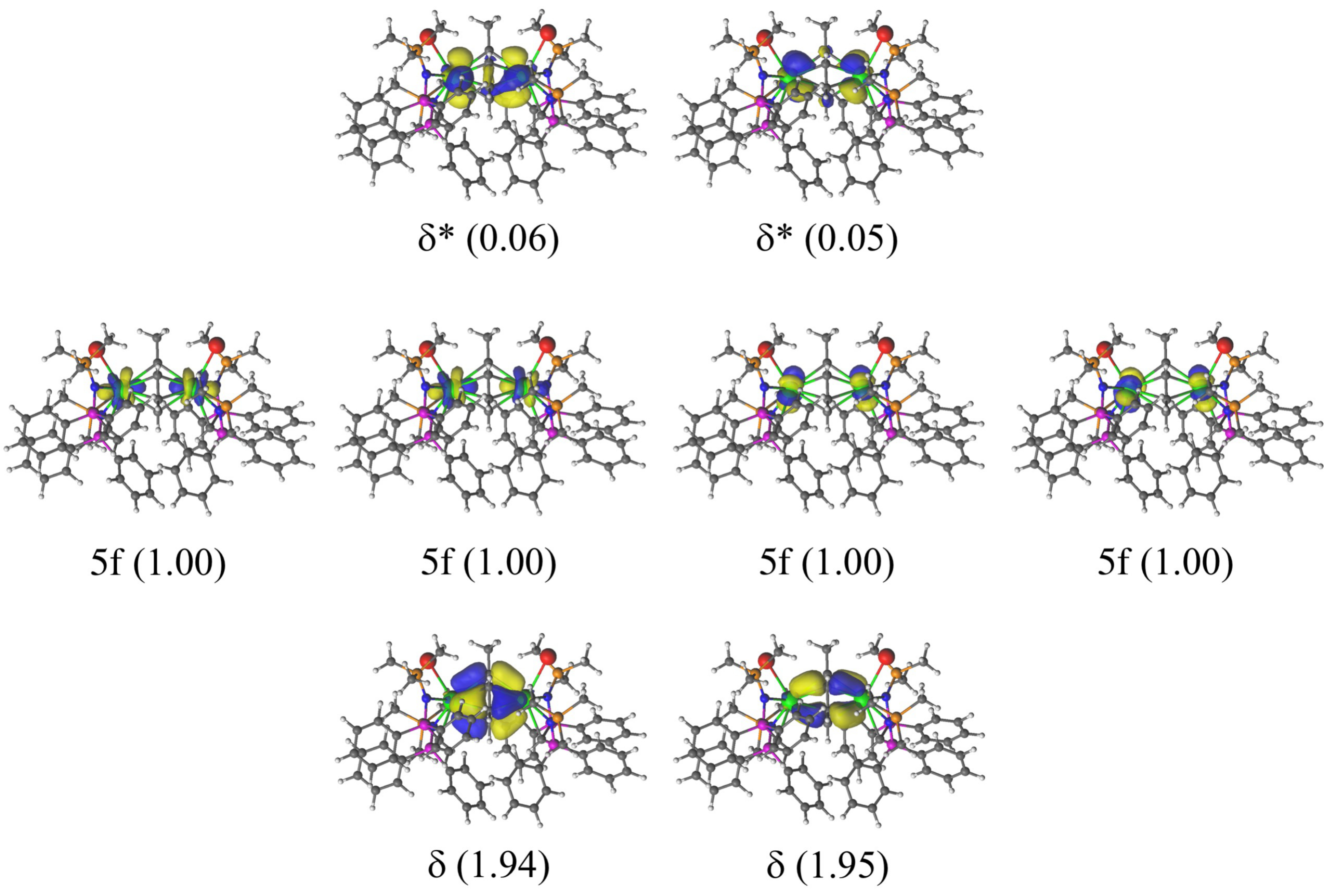


***Supplementary Figure 7.*** *The natural orbitals from the (8e,8o) active space of* ***2****. Occupation numbers are given in parentheses and the ANO-RCC basis set was used. Key: uranium, green; phosphorus, magenta; silicon, orange; iodide, pink; nitrogen, blue; carbon, gray.*

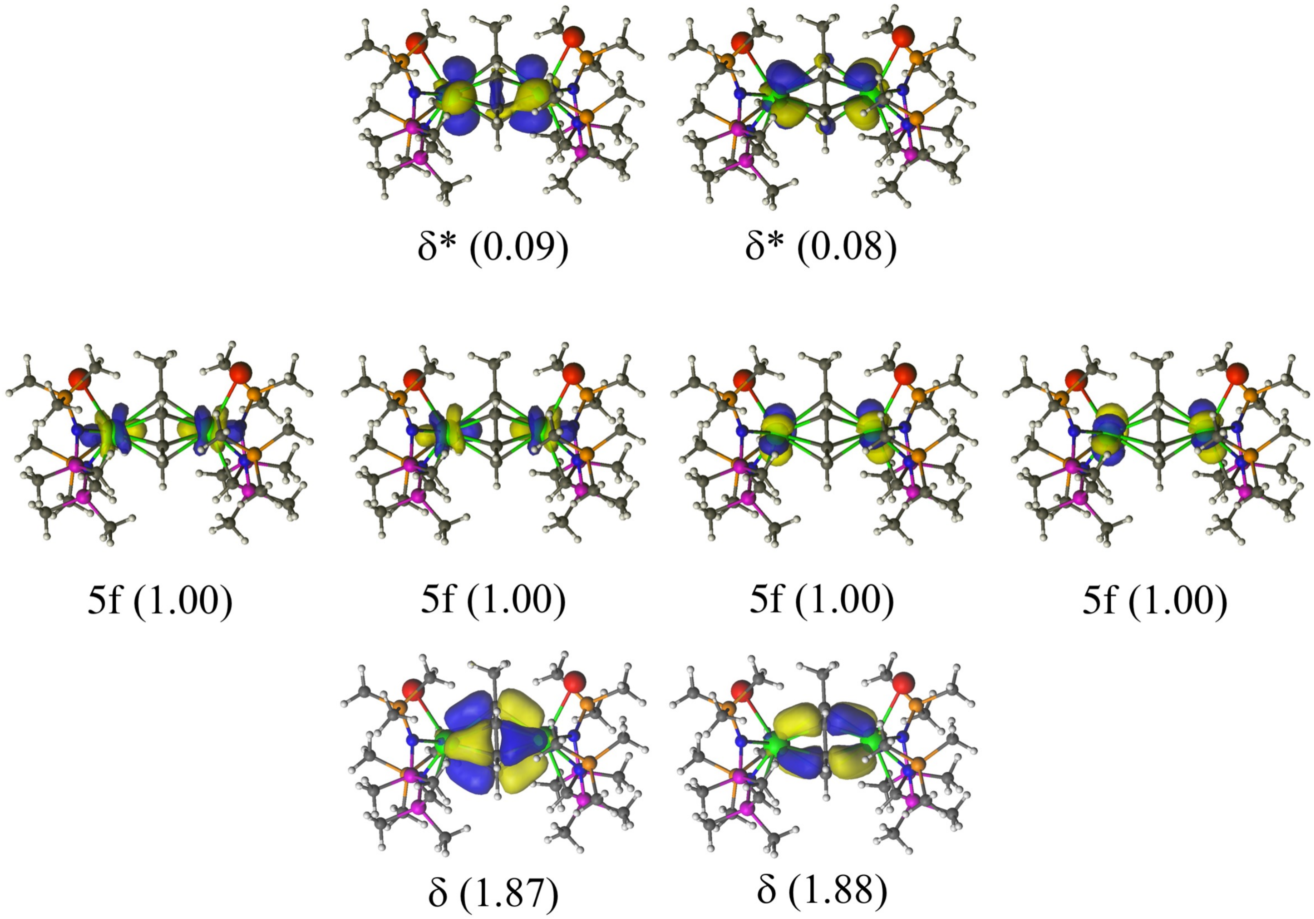


***Supplementary Figure 8.*** *The natural orbitals from the (8e,14o) active space of* ***2'****. Occupation numbers are given in parentheses and the ANO-RCC basis set was used. Orbitals with occupation numbers less than 0.03 are not plotted. Key: uranium, green; phosphorus, magenta; silicon, orange; iodide, pink; nitrogen, blue; carbon, gray.*

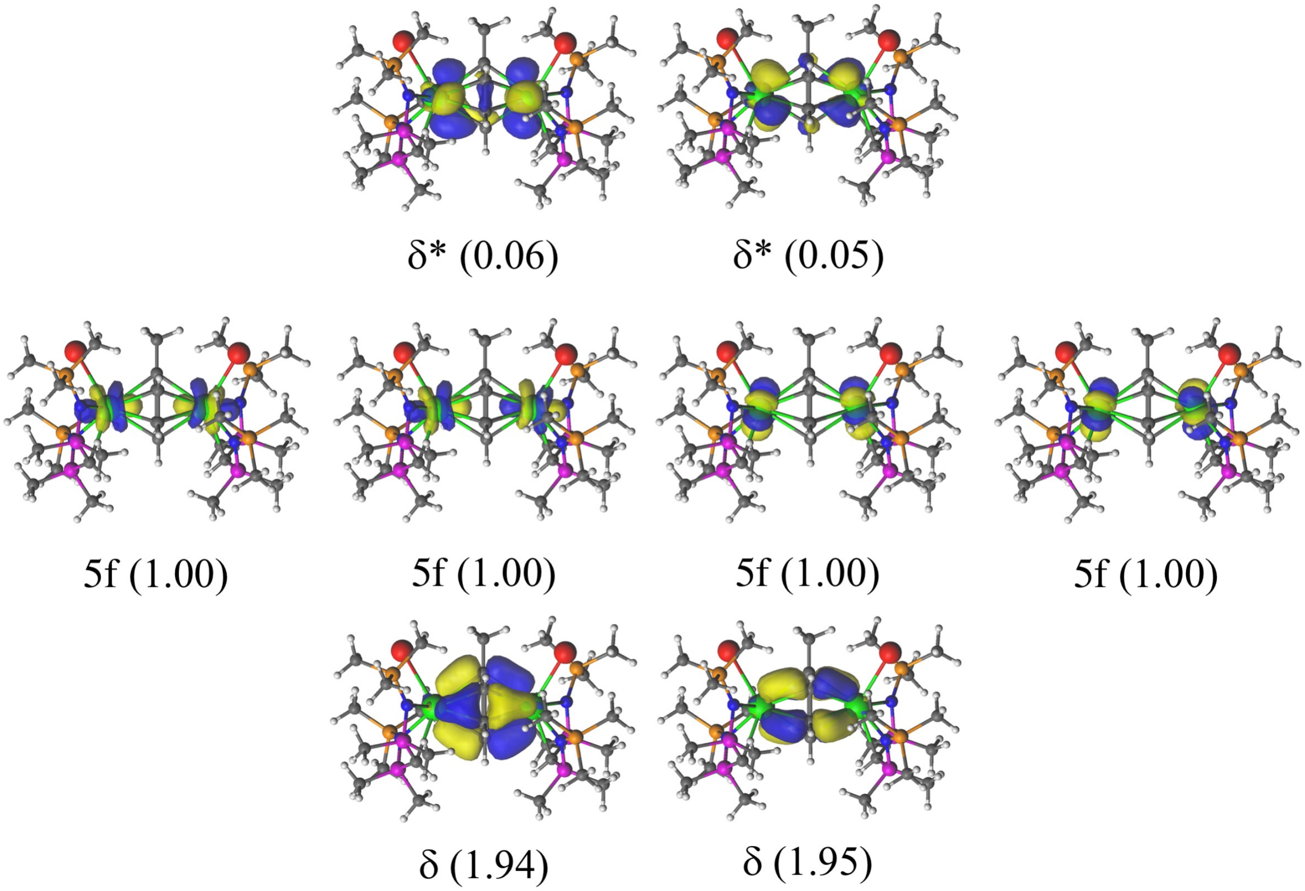


***Supplementary Figure 9.*** *The natural orbitals from the (8e,8o) active space of* ***2'.*** *Occupation numbers are given in parentheses and the ANO-RCC basis set was used. Orbitals with occupation numbers less than 0.03 are not plotted. Key: uranium, green; phosphorus, magenta; silicon, orange; iodide, pink; nitrogen, blue; carbon, gray.*

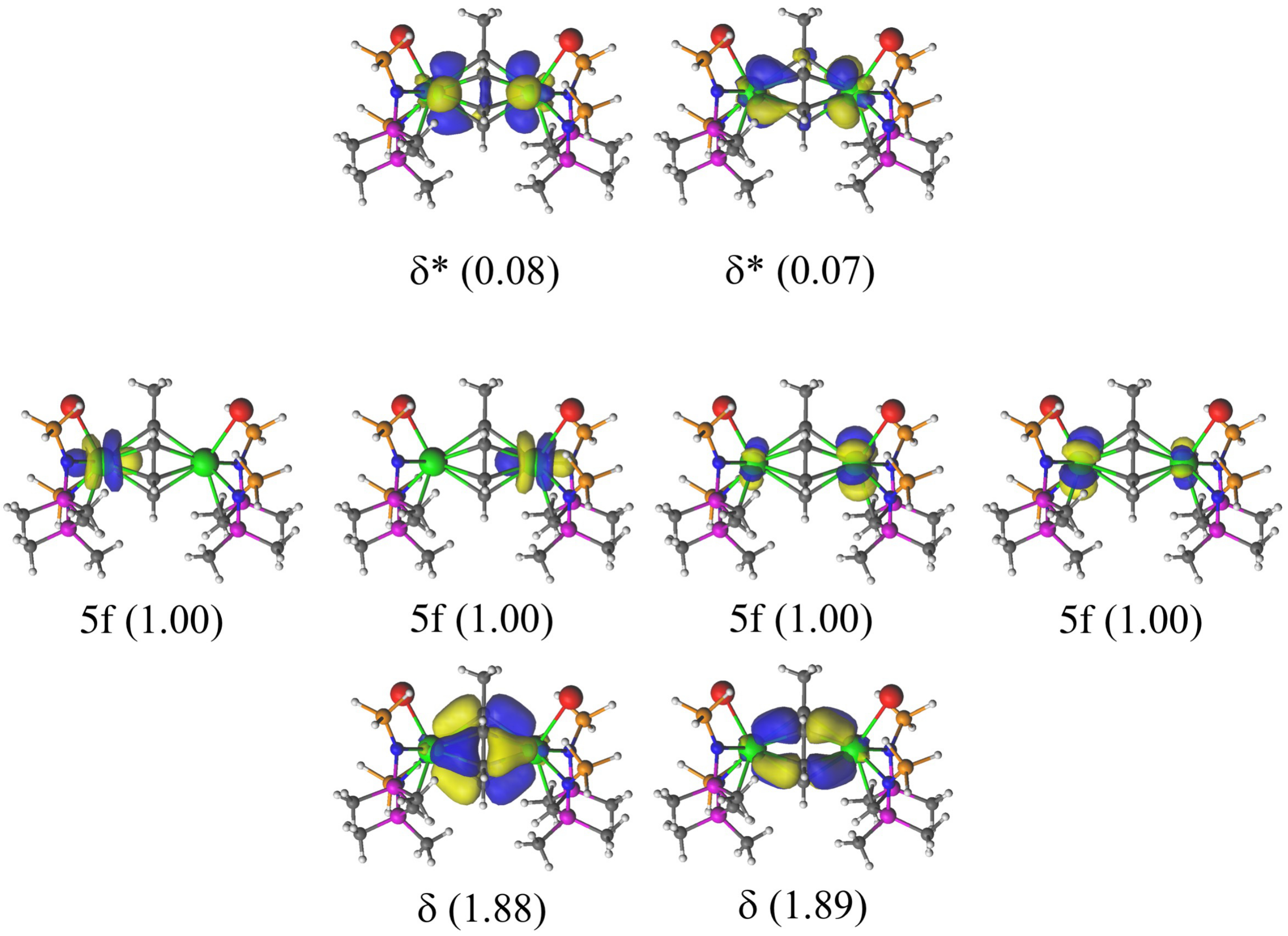


***Supplementary Figure 10.*** *The natural orbitals from the (8e,14o) active space of* ***2''****. Occupation numbers are given in parentheses and the ANO-RCC basis set was used. Orbitals with occupation numbers less than 0.03 are not plotted. Key: uranium, green; phosphorus, magenta; silicon, orange; iodide, pink; nitrogen, blue; carbon, gray.*

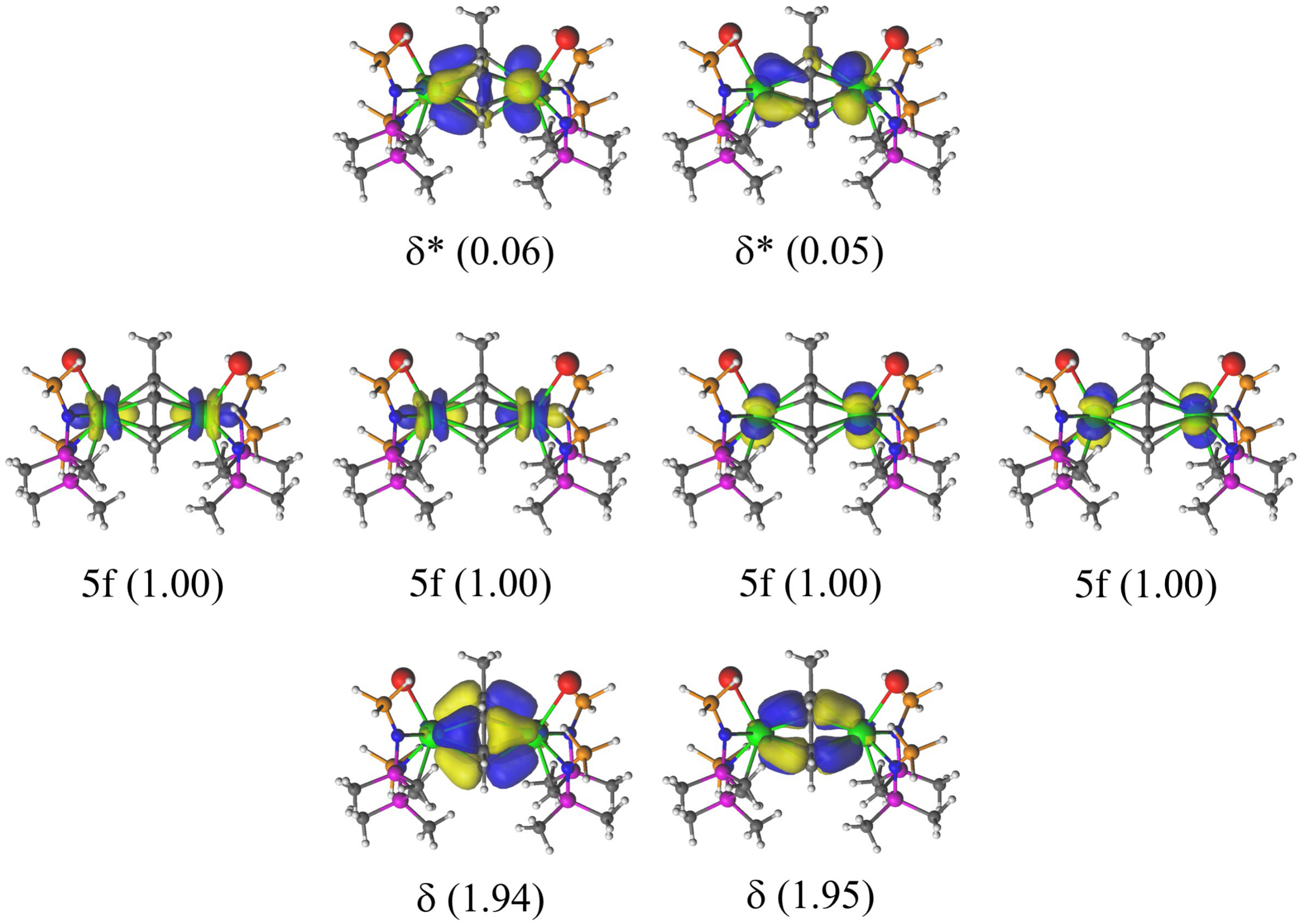


***Supplementary Figure 11.*** *The natural orbitals from the (8e,8o) active space of* ***2''****. Occupation numbers are given in parentheses and the ANO-RCC basis set was used. Key: uranium, green; phosphorus, magenta; silicon, orange; iodide, pink; nitrogen, blue; carbon, gray.*

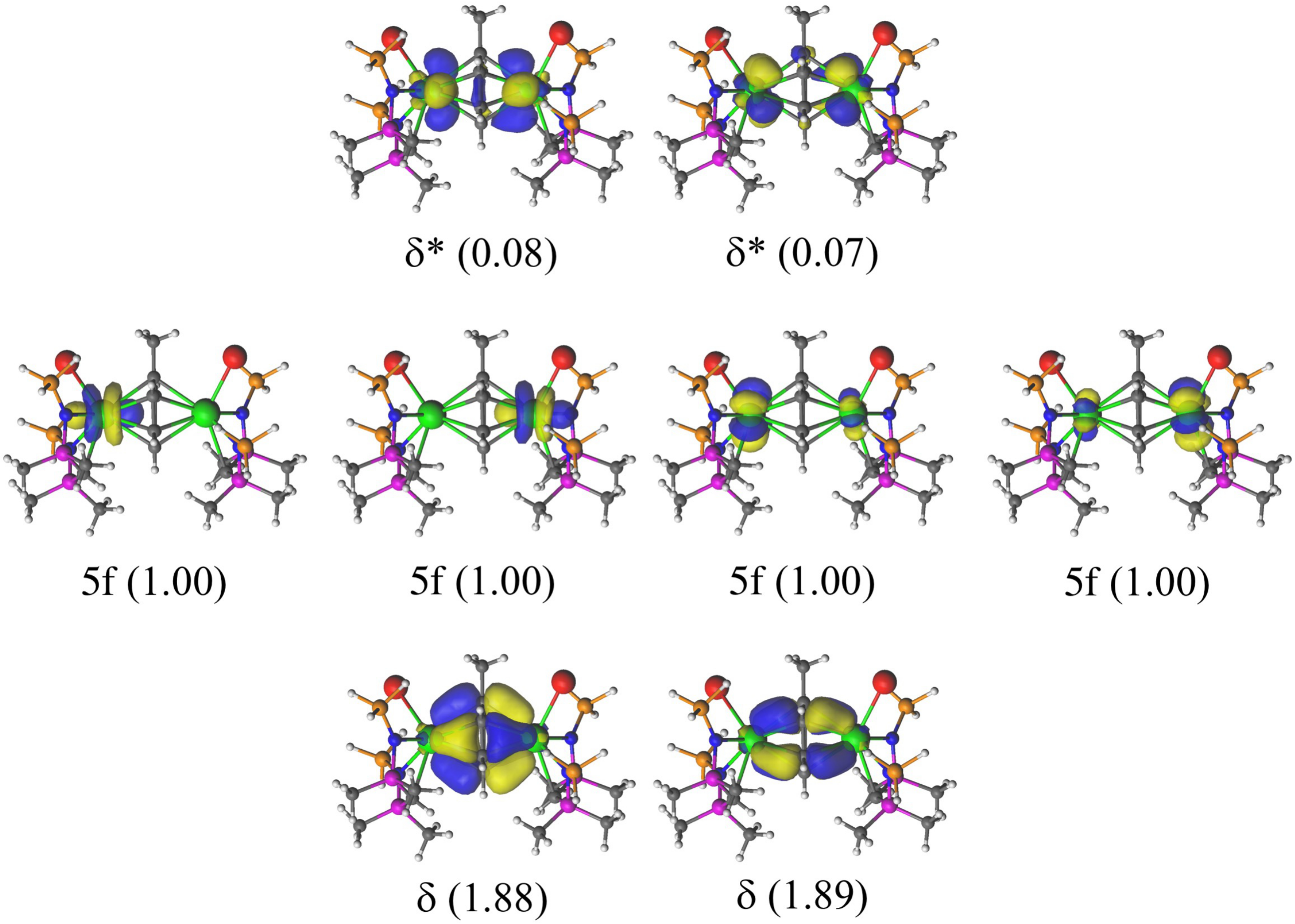


***Supplementary Figure 12.*** *The natural orbitals from the (8e,14o) active space of* ***2'''****. Occupation numbers are given in parentheses and the ANO-RCC basis set was used. Key: uranium, green; phosphorus, magenta; silicon, orange; iodide, pink; nitrogen, blue; carbon, gray.*

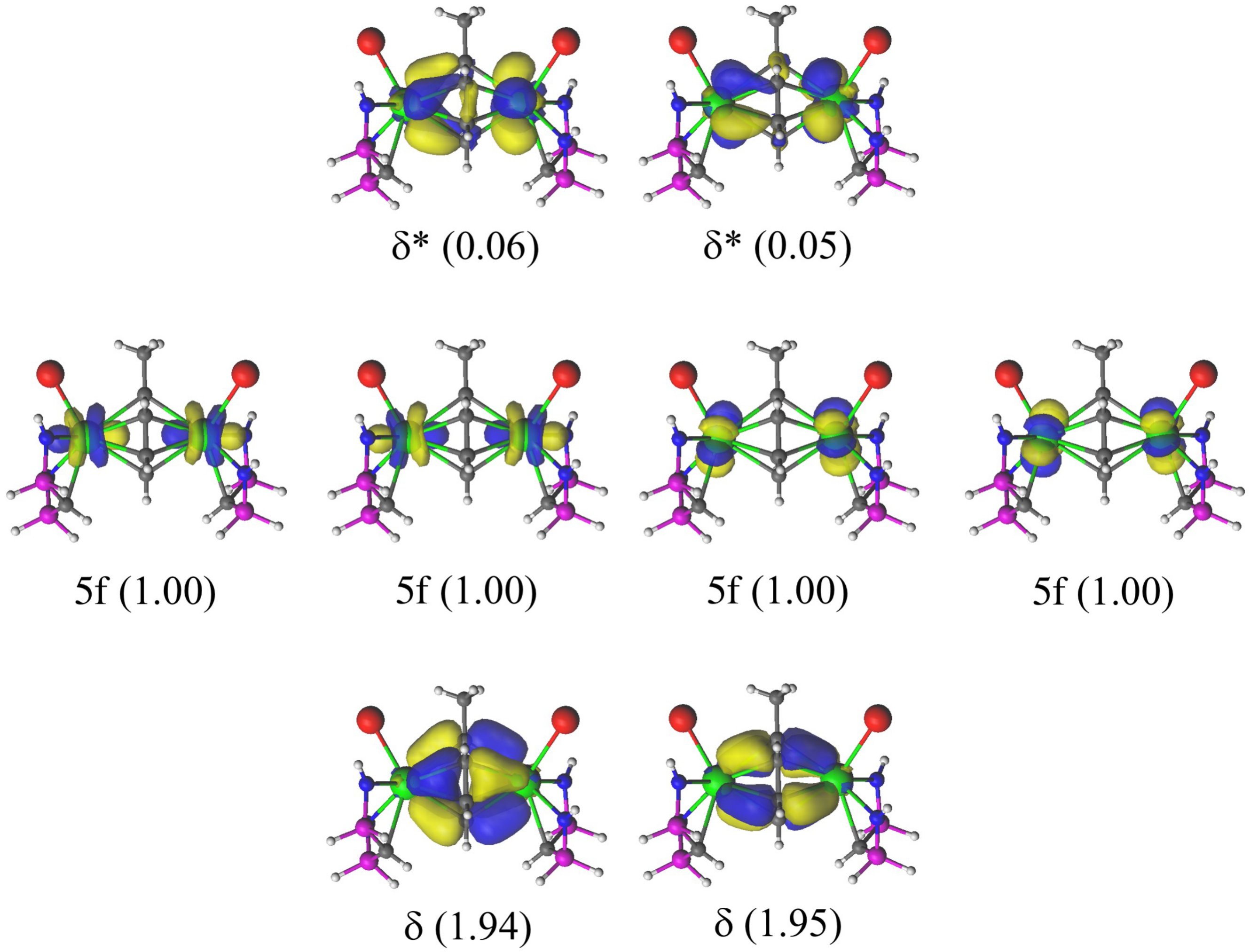


***Supplementary Figure 13.*** *The natural orbitals from the (8e,8o) active space of* ***2'''****. Occupation numbers are given in parentheses and the ANO-RCC basis set was used. Key: uranium, green; phosphorus, magenta; silicon, orange; iodide, pink; nitrogen, blue; carbon, gray.*

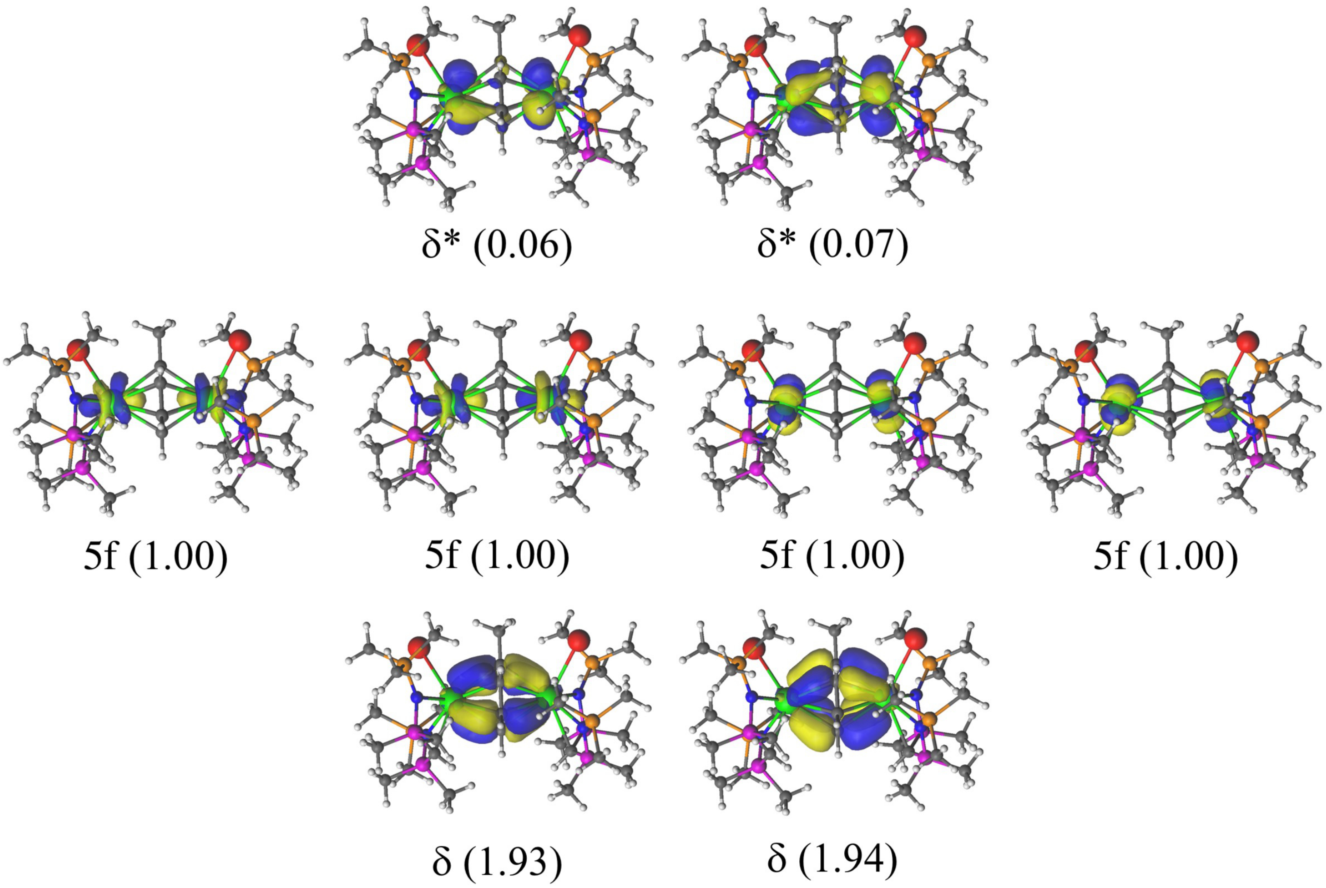


***Supplementary Figure 14.*** *The natural orbitals from the (8e,8o) active space of* ***2'.*** *Occupation numbers are given in parentheses and the ECP basis set was used. Key: uranium, green; phosphorus, magenta; silicon, orange; iodide, pink; nitrogen, blue; carbon, gray.*

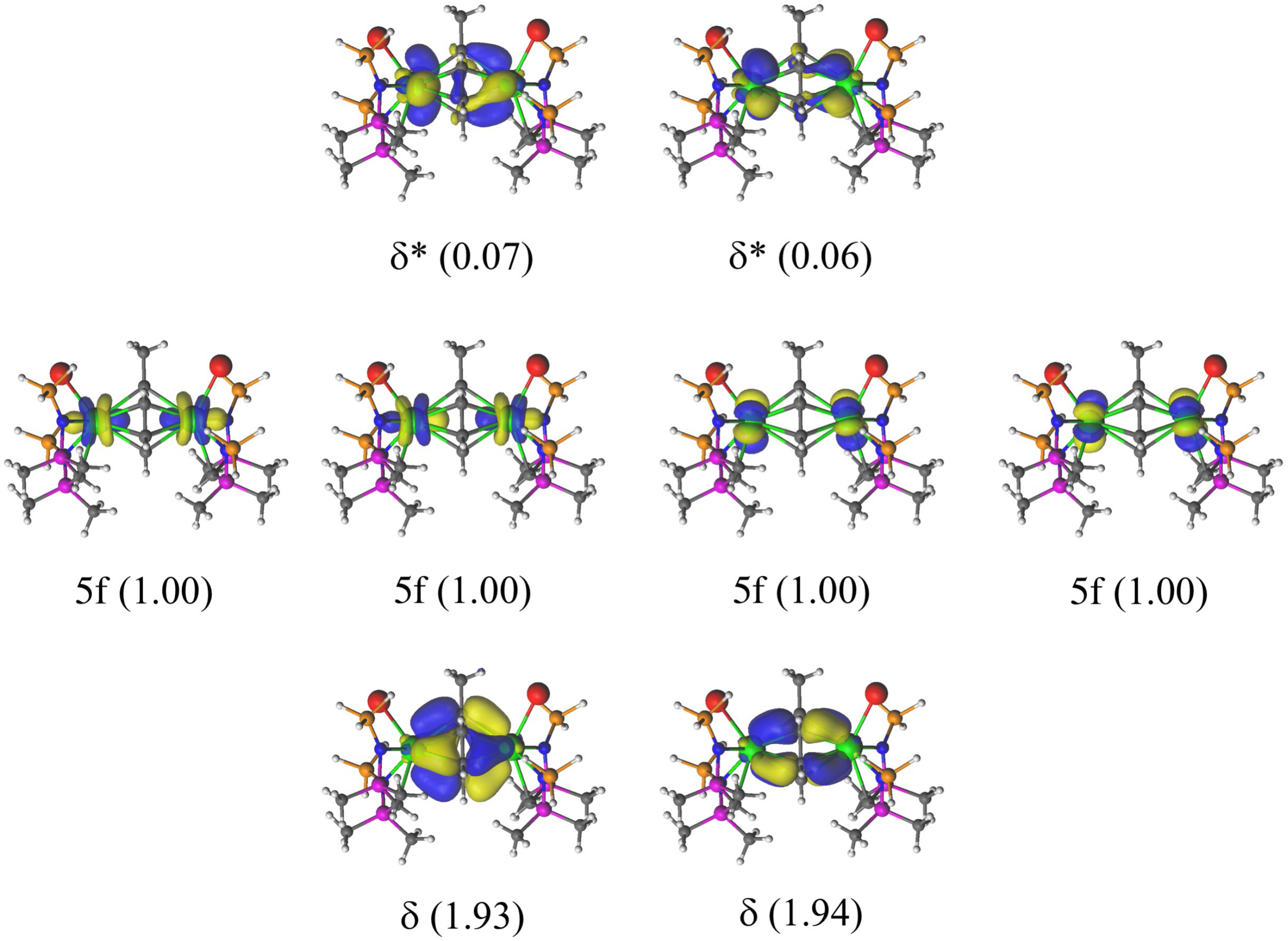


***Supplementary Figure 15.*** *The natural orbitals from the (8e,8o) active space of* ***2''****. Occupation numbers are given in parentheses and the ECP basis set was used. Key: uranium, green; phosphorus, magenta; silicon, orange; iodide, pink; nitrogen, blue; carbon, gray.*

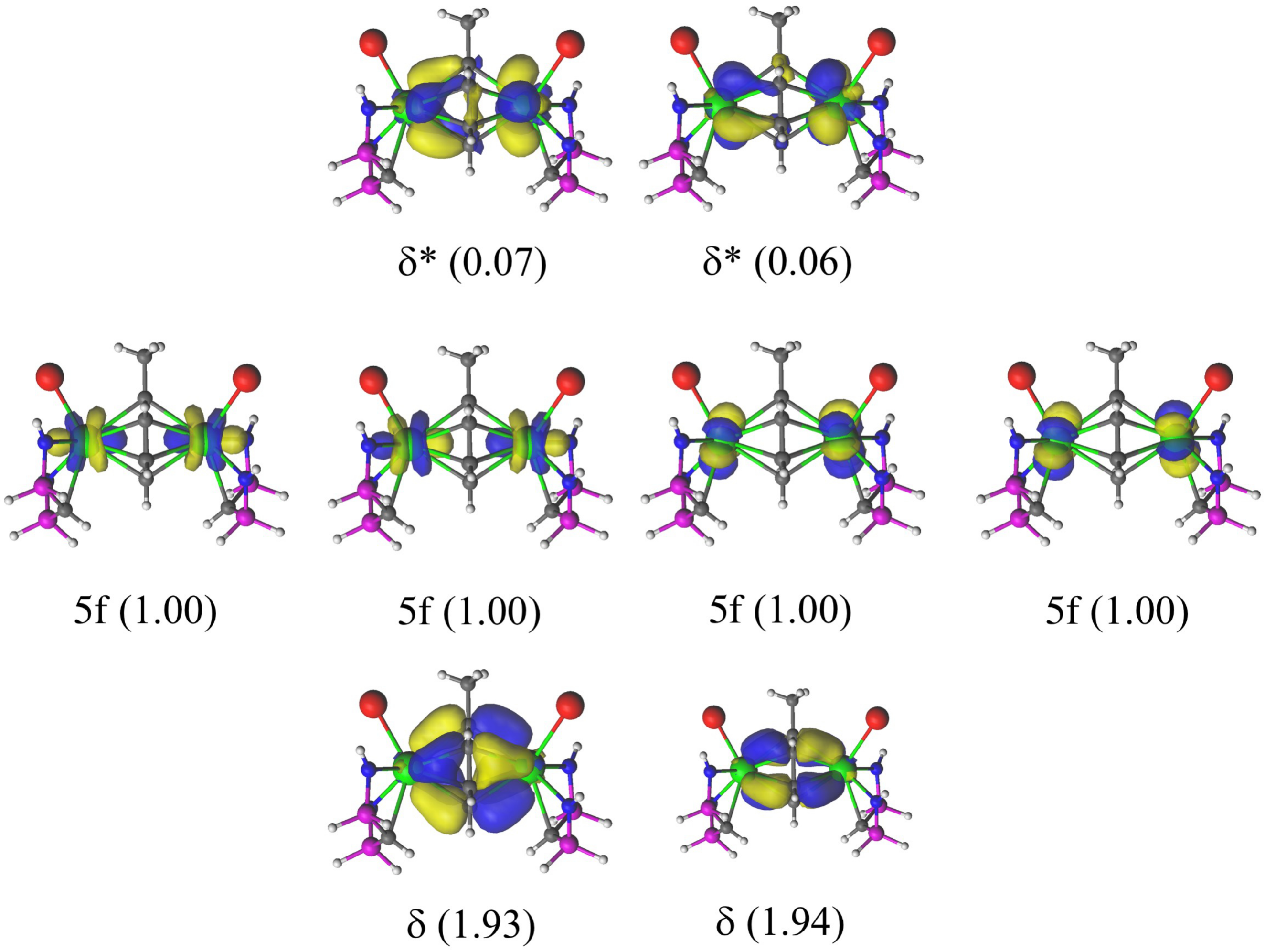


***Supplementary Figure 16.*** *The natural orbitals from the (8e,8o) active space of* ***2'''****. Occupation numbers are given in parentheses and the ECP basis set was used. Key: uranium, green; phosphorus, magenta; silicon, orange; iodide, pink; nitrogen, blue; carbon, gray.*

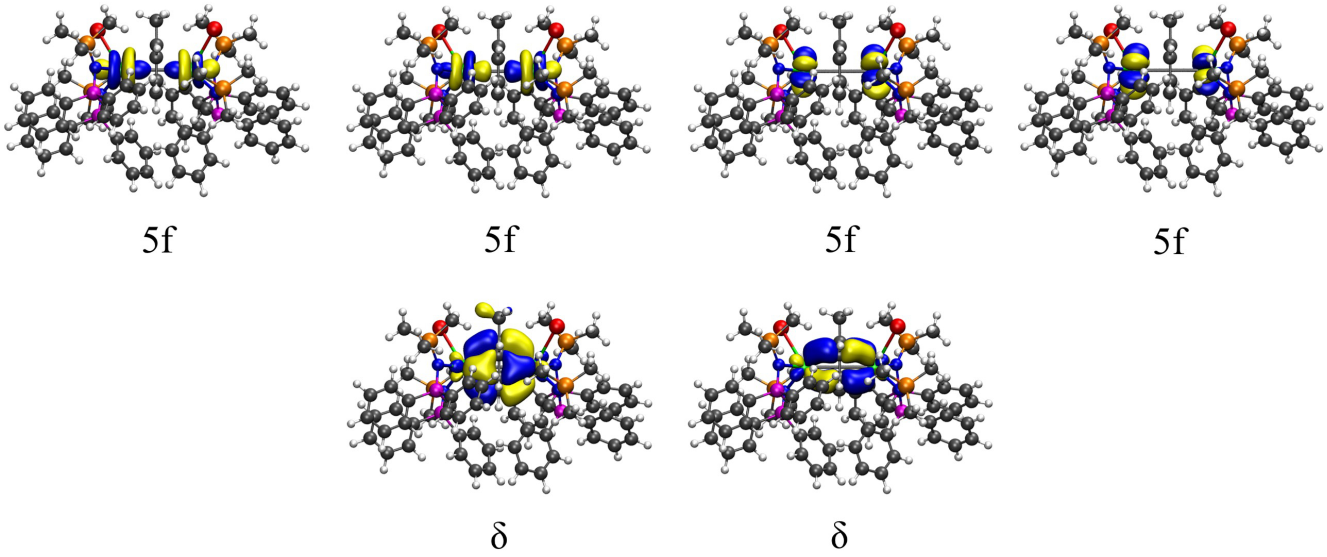


***Supplementary Figure 17.*** *The δ and 5f orbitals from the PBE/def-TZVP calculation on* **2**. *Calculations were performed using the Turbomole program package. Key: uranium, green; phosphorus, magenta; silicon, orange; iodide, pink; nitrogen, blue; carbon, gray.*

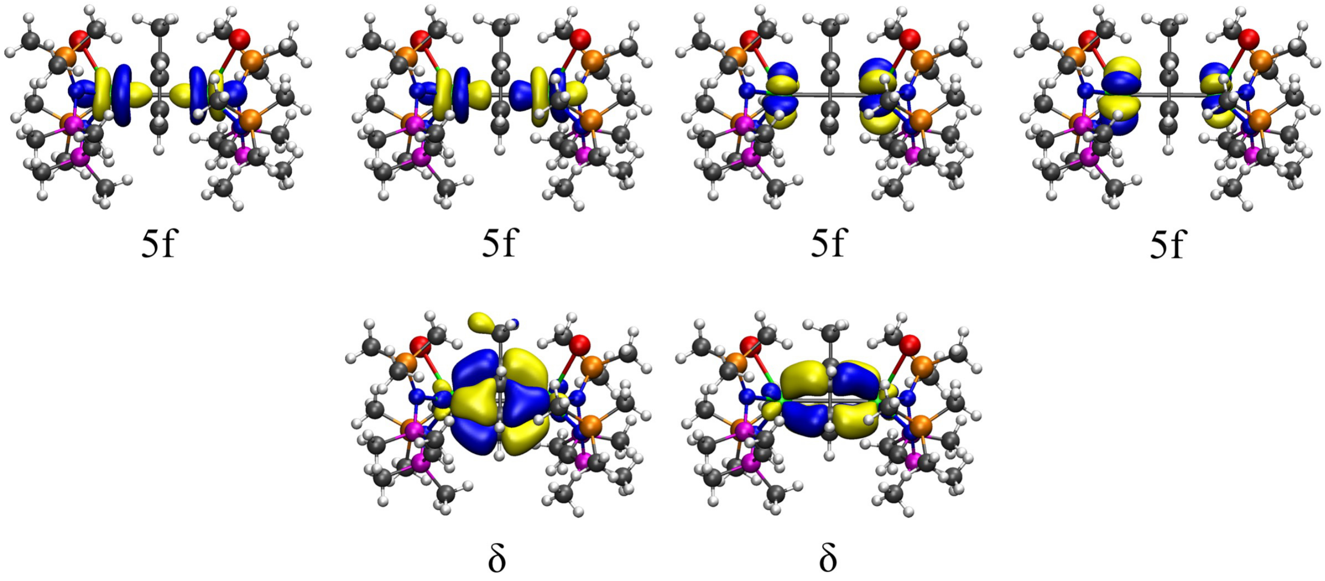


***Supplementary Figure 18.*** *The δ and 5f orbitals from the PBE/def-TZVP calculation on* ***2'****. Calculations were performed using the Turbomole program package. Key: uranium, green; phosphorus, magenta; silicon, orange; iodide, pink; nitrogen, blue; carbon, gray.*

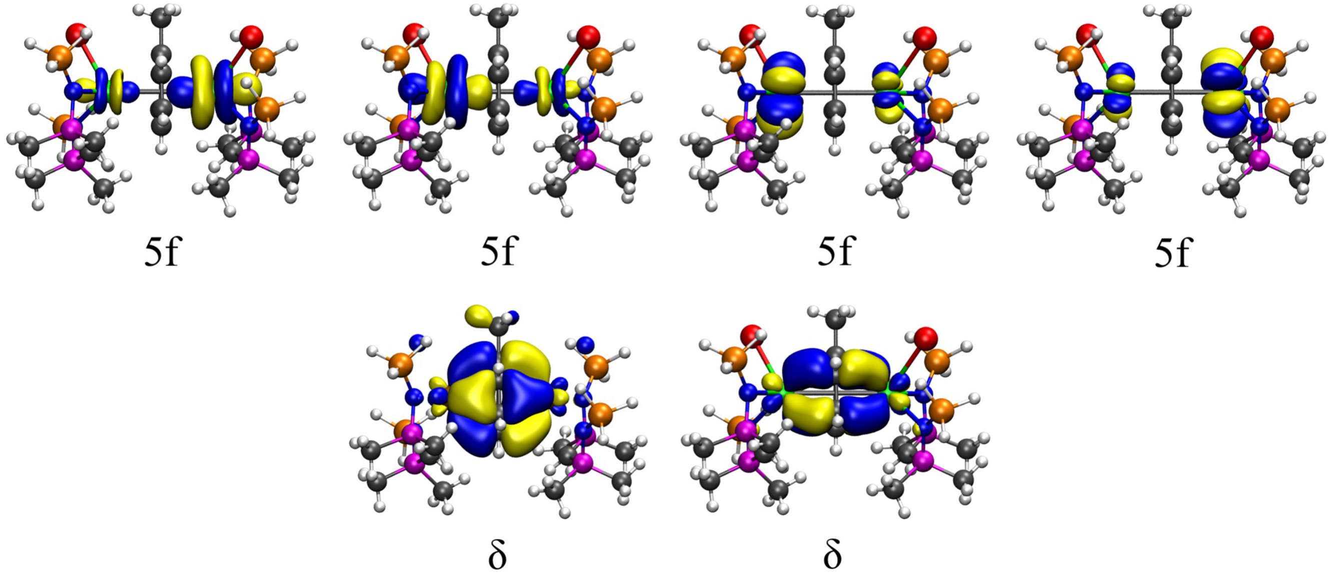


***Supplementary Figure 19.*** *The δ and 5f orbitals from the PBE/def-TZVP calculation on* ***2''****. Calculations were performed using the Turbomole program package. Key: uranium, green; phosphorus, magenta; silicon, orange; iodide, pink; nitrogen, blue; carbon, gray.*

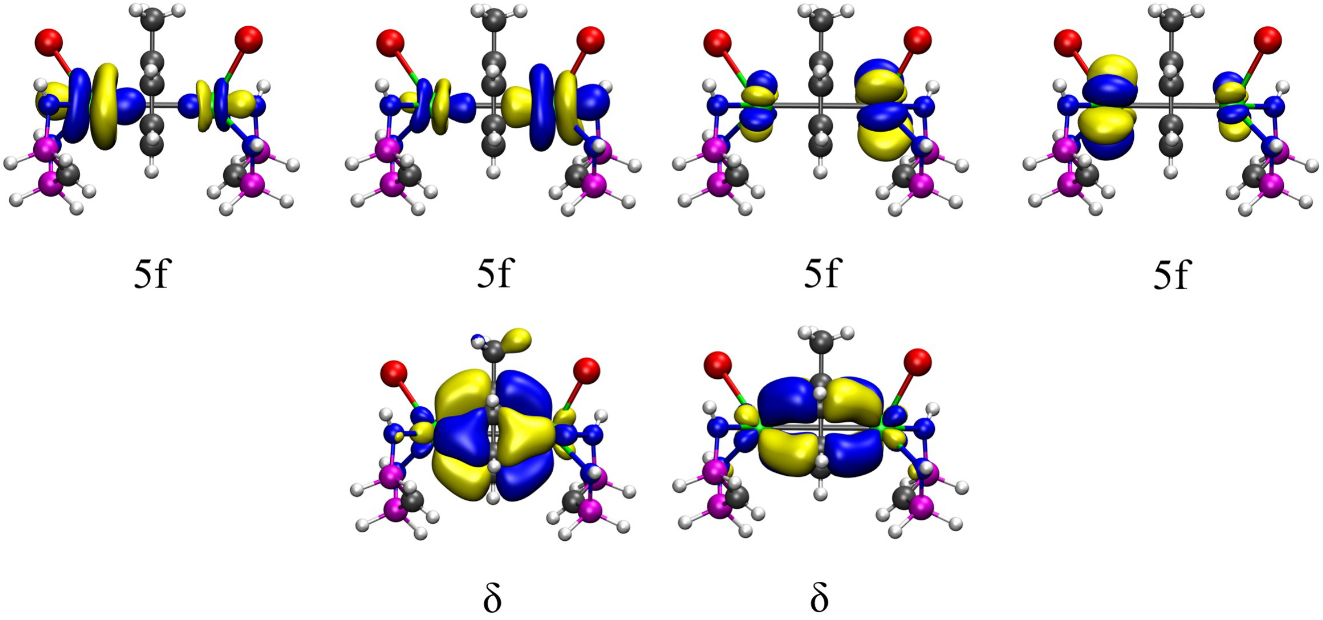


***Supplementary Figure 20.*** *The δ and 5f orbitals from the PBE/def-TZVP calculation on* ***2'''****. Calculations were performed using the Turbomole program package. Key: uranium, green; phosphorus, magenta; silicon, orange; iodide, pink; nitrogen, blue; carbon, gray.*

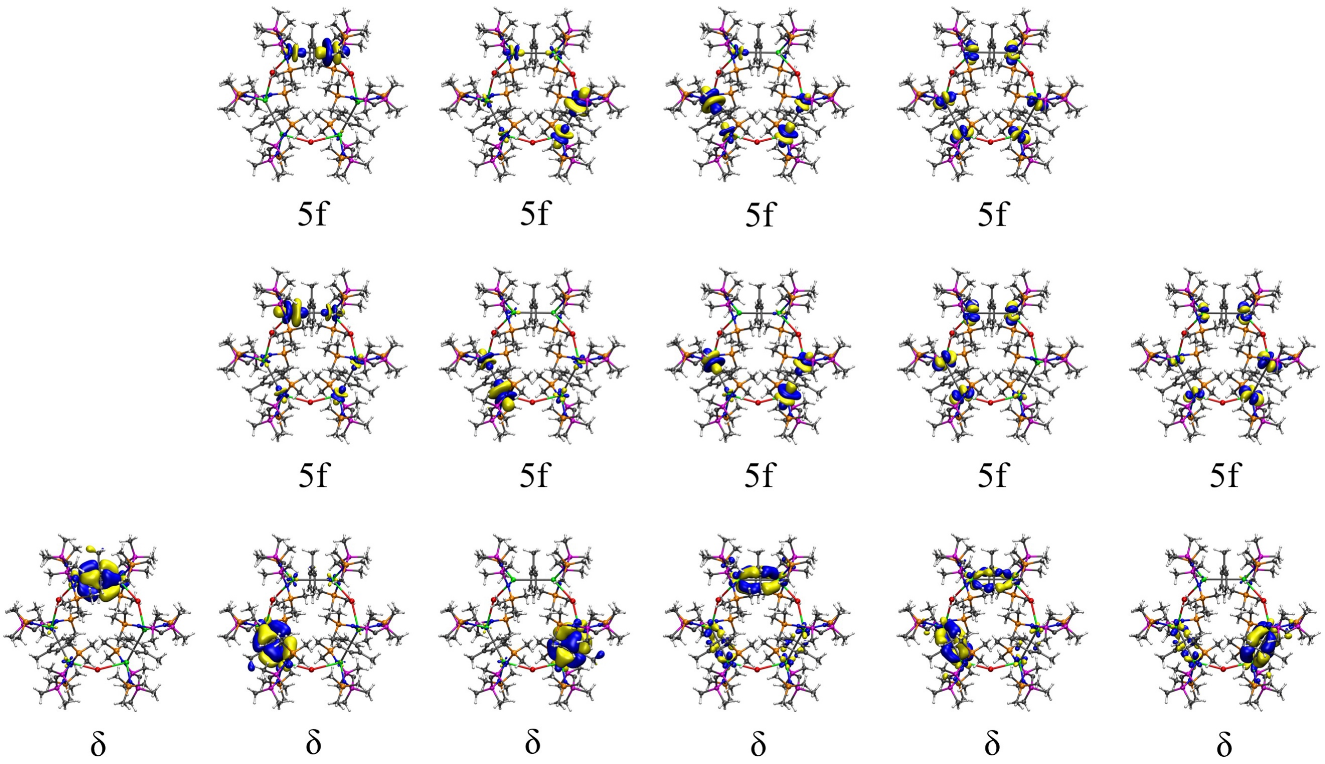


***Supplementary Figure 21.*** *The δ and 5f orbitals from the PBE/def-TZVP calculation on* ***3'****. Calculations were performed using the Turbomole program package.*

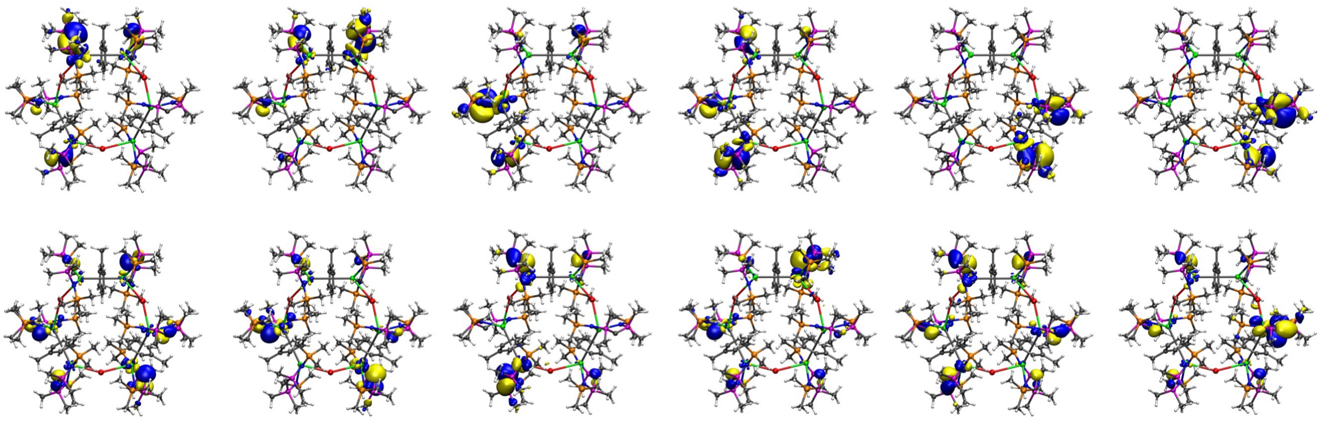

***Supplementary Figure 22.*** *The orbitals from the PBE/def-TZVP calculation on* ***3′*** *with contributions from the carbene atom. Note that the top row consists of the orbital with the largest overlap with uranium (one orbital per uranium center) while the second row consists of a more delocalized set of uranium-carbon interactions.*

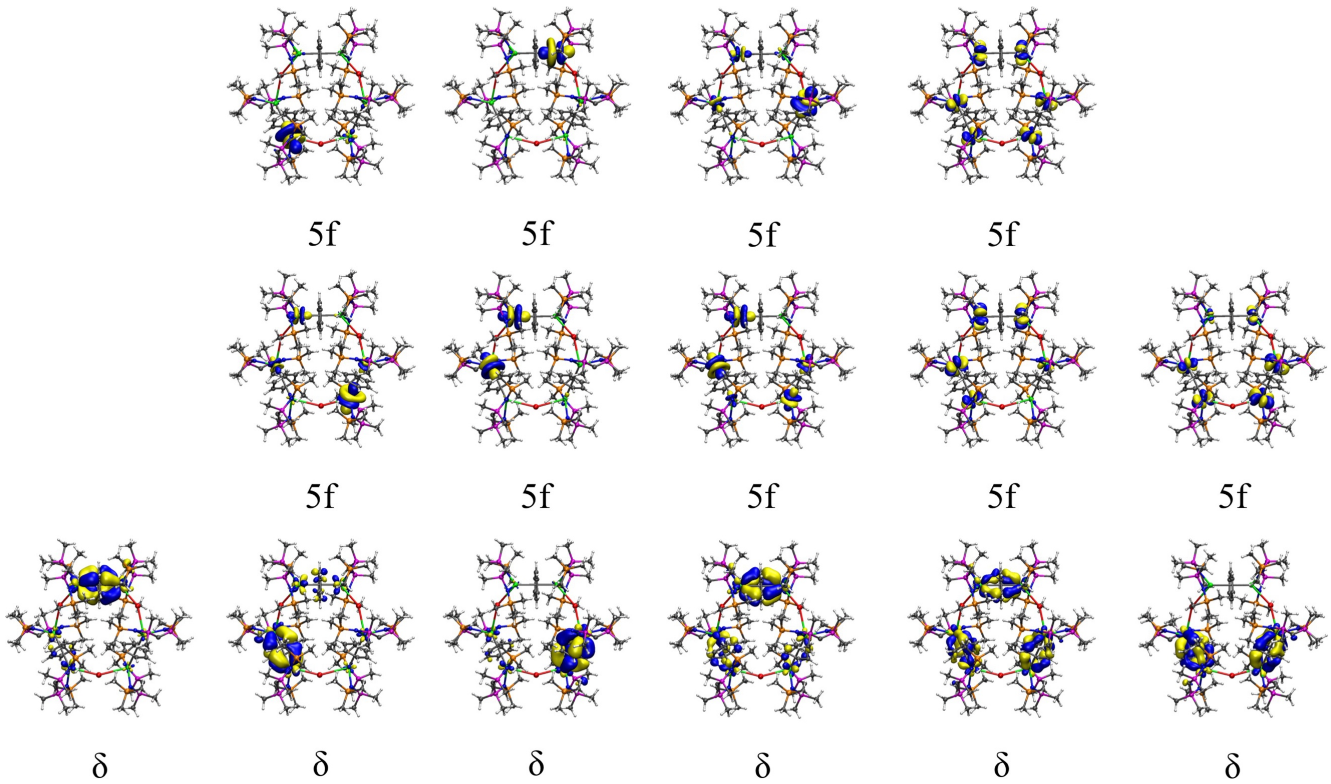


***Supplementary Figure 23.*** *The δ and 5f orbitals from the PBE/def-TZVP calculation on* ***4'****. Calculations were performed using the Turbomole program package. Key: uranium, green; phosphorus, magenta; silicon, orange; iodide, pink; nitrogen, blue; carbon, gray.*

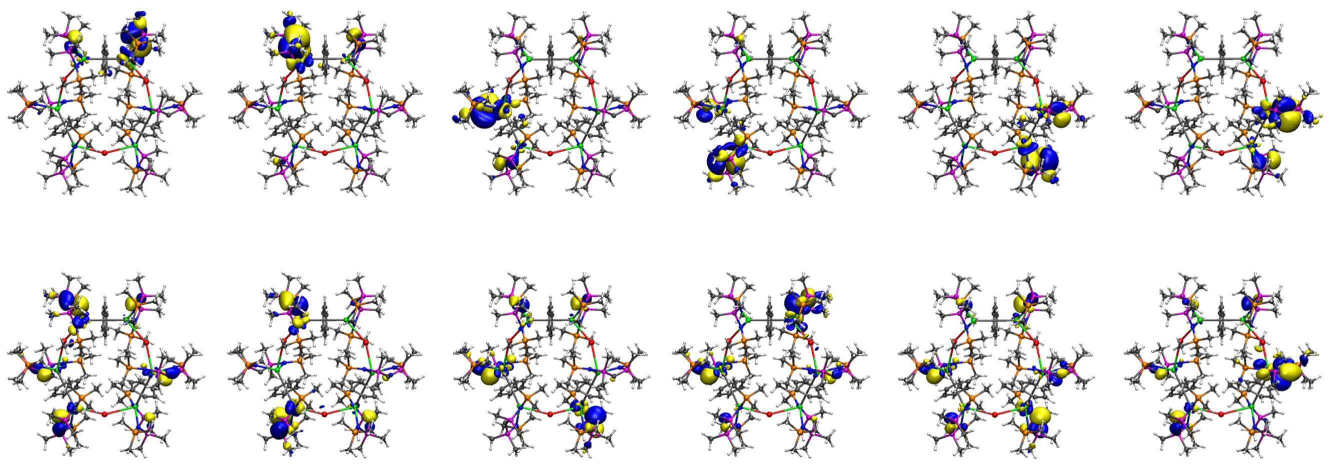

***Supplementary Figure 24.*** *The orbitals from the PBE/def-TZVP calculation on* ***4′*** *with contributions from the carbene atom. Note that the top row consists of the orbital with the largest overlap with uranium (one orbital per uranium center) while the second row consists of a more delocalized set of uranium-carbon interactions. Key: uranium, green; phosphorus, magenta; silicon, orange; iodide, pink; nitrogen, blue; carbon, gray.*

## Supplementary Tables

***Supplementary Table 1.*** *Select bond distances for* ***2*** *from experiment and DFT (Reference 41) in comparison with the PBE/def-TZVP geometry from this work.*

| | **Experiment** | **DFT (Ref. X)** | **PBE/def-TZVP** |
|---|---|---|---|
| U-N (av.) | 2.465(9) | 2.467 | 2.475 |
| U-$C_{methanide}$ (av.) | 2.753(9) | 2.776 | 2.777 |
| U-U | 4.465 | 4.375 | 4.344 |
| U-$C_{arene}$ (av.) | | 2.629 | 2.604 |
| C-C toluene ring (av.) | 1.436(16) | 1.458 | 1.450 |

***Supplementary Table 2.*** *Relative energies from the DFT calculations on **2** (PBE/def-TZVP) at the DFT optimized geometry of the quintet state. DFT $<S^2>$ values are given with their ideal values in parentheses. Both the closed shell (CS) and broken symmetry (BS) singlets are computed.*

| **Spin State** | **ΔE PBE/def-TZVP (kcal/mol)** | **$<S^2>$** |
|---|---|---|
| CS singlet | 34.9 | 0.00 (0) |
| BS singlet | 14.7 | 3.14 (1) |
| triplet | 6.4 | 3.14 (2) |
| quintet | 0.0 | 6.08 (6) |

***Supplementary Table 3.*** *CASSCF and CASPT2 relative energies in kcal.mol$^{-1}$ for **2** with the ANO-RCC basis set. Comparison of the two active spaces is also given.*

| | | (8e,14o) | | (8e,8o) | |
|---|---|---|---|---|---|
| | **State** | **CASSCF** | **CASPT2** | **CASSCF** | **CASPT2** |
| **2** | $^{1}A$ | 0 | 0 | 0 | 0 |
| | $^{3}A$ | 0.77 | 0.03 | 0.00 | 0.03 |
| | $^{5}A$ | 2.31 | 2.41 | 1.63 | 2.46 |

***Supplementary Table 4.*** *Charge analysis based on CASSCF calculations on the quintet state of* ***2****. The ANO-RCC basis set was used and both LoProp and Mulliken charges were computed.*

| Active Space | Charge Model | U (av.) | Arene (sum) | P (av.) | N (av.) | Methanide C (av.) |
|---|---|---|---|---|---|---|
| (8e,14o) | LoProp | 2.49 | -2.41 | 1.20 | -1.12 | -0.91 |
| (8e,8o) | LoProp | 2.58 | -2.53 | 1.20 | -1.12 | -0.91 |
| (8e,14o) | Mulliken | 2.68 | -0.22 | 0.98 | -1.30 | -0.91 |
| (8e,8o) | Mulliken | 2.84 | -0.26 | 0.98 | -1.30 | -0.91 |

***Supplementary Table 5.*** *CASSCF and CASPT2 relative energies in kcal.mol$^{-1}$ for* ***2, 2', 2''***, *and* ***2'''*** *with the ANO-RCC basis set. Comparison of the two active spaces is also given*

| | | **(8e,14o)** | | **(8e,8o)** | |
|---|---|---|---|---|---|
| | **State** | **CASSCF** | **CASPT2** | **CASSCF** | **CASPT2** |
| **2** | $^1$A | 0 | 0 | 0 | 0 |
| | $^3$A | 0.77 | 0.03 | 0.00 | 0.03 |
| | $^5$A | 2.31 | 2.41 | 1.63 | 2.46 |
| **2'** | $^1$A | 0 | 0 | 0 | 0 |
| | $^3$A | 0.76 | 1.53 | 0.85 | 1.71 |
| | $^5$A | 2.35 | 3.92 | 2.50 | 4.21 |
| **2''** | $^1$A | 0 | 0 | 0 | 0 |
| | $^3$A | 0.75 | 1.34 | 0.85 | 1.61 |
| | $^5$A | 2.26 | 3.90 | 2.45 | 3.96 |
| **2'''** | $^1$A | 0 | 0 | 0 | 0 |
| | $^3$A | 0.73 | 1.22 | 0.83 | 1.49 |
| | $^5$A | 2.18 | 3.62 | 2.41 | 3.43 |

***Supplementary Table 6.*** *Charge analysis based on CASSCF calculations on the quintet state of* ***2, 2', 2'',*** *and* ***2'''****. The ANO-RCC basis set was used and both LoProp and Mulliken charges were computed.*

| | **Active Space** | **Charge Model** | **U (av.)** | **Arene (sum)** | **P (av.)** | **N (av.)** | **Methanide C (av.)** |
|---|---|---|---|---|---|---|---|
| **2** | (8*e*,14*o*) | LoProp | 2.49 | -2.41 | 1.20 | -1.12 | -0.91 |
| | (8*e*,8*o*) | LoProp | 2.58 | -2.53 | 1.20 | -1.12 | -0.91 |
| | (8*e*,14*o*) | Mulliken | 2.68 | -0.22 | 0.98 | -1.3 | -0.91 |
| | (8*e*,8*o*) | Mulliken | 2.84 | -0.26 | 0.98 | -1.3 | -0.91 |
| **2'** | (8*e*,14*o*) | LoProp | 2.50 | -2.46 | 1.27 | -1.14 | -0.95 |
| | (8*e*,8*o*) | LoProp | 2.53 | -2.60 | 1.27 | -1.14 | -0.96 |
| | (8*e*,14*o*) | Mulliken | 1.91 | -1.13 | 1.41 | -1.14 | -0.65 |
| | (8*e*,8*o*) | Mulliken | 1.90 | -1.03 | 1.41 | -1.14 | -0.62 |
| **2''** | (8*e*,14*o*) | LoProp | 2.60 | -2.28 | 1.39 | -1.20 | -1.12 |
| | (8*e*,8*o*) | LoProp | 2.65 | -2.37 | 1.39 | -1.20 | -1.12 |
| | (8*e*,14*o*) | Mulliken | 1.55 | -1.14 | 1.18 | -0.89 | -0.71 |
| | (8*e*,8*o*) | Mulliken | 1.55 | -1.46 | 1.18 | -0.89 | -0.71 |
| **2'''** | (8*e*,14*o*) | LoProp | 2.56 | -2.22 | 1.16 | -1.12 | -1.02 |
| | (8*e*,8*o*) | LoProp | 2.61 | -2.30 | 1.16 | -1.12 | -1.02 |
| | (8*e*,14*o*) | Mulliken | 1.58 | -1.80 | 0.83 | -0.65 | -0.66 |
| | (8*e*,8*o*) | Mulliken | 1.58 | -1.10 | 0.83 | -0.65 | -0.65 |

***Supplementary Table 7.*** *CASSCF and CASPT2 relative energies in kcal.mol$^{-1}$ for* ***2, 2', 2''***, *and* ***2'''*** *with the ANO-RCC and ECP basis set. The (8e,8o) active space is used for both basis sets.*

| | | ANO-RCC | | Dolg ECP | |
|---|---|---|---|---|---|
| | **State** | **CASSCF** | **CASPT2** | **CASSCF** | **CASPT2** |
| **2'** | $^1$A | 0 | 0 | 0 | 0 |
| | $^3$A | 0.85 | 1.53 | 0.95 | 1.41 |
| | $^5$A | 2.50 | 3.94 | 2.78 | 3.78 |
| **2''** | $^1$A | 0 | 0 | 0 | 0 |
| | $^3$A | 0.85 | 1.71 | 0.93 | 1.46 |
| | $^5$A | 2.45 | 4.21 | 2.68 | 3.42 |
| **2'''** | $^1$A | 0 | 0 | 0 | 0 |
| | $^3$A | 0.83 | 1.49 | 0.91 | 1.45 |
| | $^5$A | 2.41 | 3.43 | 2.64 | 3.53 |

***Supplementary Table 8.*** *Charge analysis based on CASSCF calculations on the quintet state of* ***2'***, ***2''***, *and* ***2'''***. *Mulliken chargers are reported for the ANO-RCC and Dolg ECP basis sets.*

| | **Basis Set** | **U (av.)** | **Arene (sum)** | **P (av.)** | **N (av.)** | **Methanide C (av.)** |
|---|---|---|---|---|---|---|
| **2'** | ANO-RCC | 1.90 | -1.03 | 1.41 | -1.14 | -0.62 |
| | ECP | 1.56 | -0.97 | 1.28 | -1.38 | -1.13 |
| **2''** | ANO-RCC | 1.55 | -1.46 | 1.18 | -0.89 | -0.71 |
| | ECP | 1.38 | -0.83 | 1.19 | -1.40 | -1.07 |
| **2'''** | ANO-RCC | 1.58 | -1.10 | 0.83 | -0.65 | -0.65 |
| | ECP | 1.30 | -1.70 | 0.98 | -1.15 | -1.02 |

***Supplementary Table 9.*** *RASSCF energies for* ***4'''*** *in kcal/mol with a (21,2,2;6,11,6) active space and the Dolg-ECP basis set.*

| **State Computed** | **RASSCF** |
|---|---|
| $^{8}$A | 0.02 |
| $^{8}$B | 0.02 |
| $^{10}$A | 0.00 |
| $^{10}$B | 10.00 |
| $^{12}$A | 39.51 |
| $^{12}$B | 28.31 |
| $^{14}$A | 51.92 |
| $^{14}$B | 39.02 |

***Supplementary Table 10.*** *CASPT2 relative energies in kcal/mol for* ***4'''*** *with a (9e, 17o) active space and the Dolg-ECP basis set.*

| **Multiplicity** | **CASPT2** |
|---|---|
| Doublet | 0.00 |
| Quartet | 0.14 |
| Sextet | 0.39 |
| Octet | 0.31 |
| Decet | 0.69 |

***Supplementary Table 11.*** *Mulliken charge analysis of the quintet state for* ***2'''*** *and the S=9/2 state for* ***4'''****. An (8e, 8o) active space was used for* ***2'''*** *while* ***4'''*** *was studied using RASSCF. For* ***4'''****, each U-arene-U group is reported separately.*

| | **Basis** | **U (av.)** | **Arene (sum)** | **P (av.)** | **N (av.)** | **Methanide C (av.)** |
|---|---|---|---|---|---|---|
| **2'''** | ECP | 1.30 | -1.70 | 0.98 | -1.15 | -1.02 |
| **4'''** | ECP | 1.45 | -0.64 | 0.94 | -1.17 | -0.85 |
| | | 1.49 | -0.65 | 0.96 | -1.17 | -0.90 |
| | | 1.50 | -0.65 | 0.96 | -1.18 | -0.89 |

***Supplementary Table 12.*** *Natural population analysis (NPA) charges were computed for the quintet state for **2** and its models. Energy calculations were performed with the Turbomole program package for PBE/def-TZVP.*

| | U (av.) | Arene (sum) | P (av.) | N (av.) | Methanide C (av.) |
|---|---|---|---|---|---|
| **2** | 0.62 | -0.47 | 1.76 | -1.39 | -1.24 |
| **2'** | 0.63 | -0.53 | 1.75 | -1.26 | -1.26 |
| **2''** | 0.41 | -0.48 | 1.80 | -1.37 | -1.25 |
| **2'''** | 0.39 | -0.49 | 1.31 | -1.14 | -1.21 |

***Supplementary Table 13.*** *Natural population analysis (NPA) charges were computed for the S=9/2 state of **3'**, **4'** and **4'''**. Energy calculations were performed with the Turbomole program package for PBE/def-TZVP. Chargers are reported for each U-arene-U group separately.*

| | U (av.) | Arene (sum) | P (av.) | N (av.) | Methanide C (av.) |
|---|---|---|---|---|---|
| **3'** | 0.54 | -0.30 | 1.81 | -1.41 | -1.30 |
| | 0.54 | -0.29 | 1.81 | -1.41 | -1.31 |
| | 0.54 | -0.30 | 1.81 | -1.41 | -1.31 |
| **4'** | 0.54 | -0.29 | 1.81 | -1.41 | -1.31 |
| | 0.54 | -0.29 | 1.81 | -1.41 | -1.30 |
| | 0.54 | -0.29 | 1.81 | -1.41 | -1.30 |
| **4'''** | 0.50 | -0.29 | 1.31 | -1.14 | -1.23 |
| | 0.54 | -0.30 | 1.33 | -1.13 | -1.28 |
| | 0.50 | -0.27 | 1.31 | -1.14 | -1.23 |

***Supplementary Table 14.*** *MDC-q charge analysis of* ***3'*** *and* ***4'****. Each U-arene-U group is listed separately. Calculations were performed using the ADF software package with PBE/TZ2P.*

| | **U (av.)** | **Arene (sum)** | **P (av.)** | **N (av.)** | **$C_{carbene}$ (av.)** |
|---|---|---|---|---|---|
| **3'** | 1.19 | -1.21 | 1.22 | -1.04 | -0.98 |
| | 1.19 | -1.19 | 1.22 | -1.04 | -0.97 |
| | 1.19 | -1.22 | 1.22 | -1.03 | -0.98 |
| **4'** | 1.18 | -1.08 | 1.21 | -1.03 | -0.96 |
| | 1.17 | -1.07 | 1.20 | -1.03 | -0.98 |
| | 1.18 | -1.08 | 1.20 | -1.04 | -0.95 |

## Supplementary Methods

### *General Procedures*

All manipulations were carried out using standard Schlenk and glove box techniques under an atmosphere of dry nitrogen. Toluene was dried by passage through an activated alumina tower and benzene was dried by refluxing over potassium. Both solvents were stored over potassium mirrors and degassed before use. Deuterated solvents were distilled from potassium, degassed by three freeze-pump-thaw cycles and stored under nitrogen. The compounds $[\{U(BIPM^{TMS})(\mu\text{-}I)(I)\}_2]$ (**1**) and $KC_8$ were prepared according to literature procedures.[1,2] $^1H$ NMR spectra were recorded on a spectrometer operating at 400.13 MHz; chemical shifts are quoted in ppm and are relative to TMS. FTIR spectra were recorded as Nujol mulls in KBr discs on a Bruker Tensor 27 spectrometer. UV/Vis/NIR spectra were recorded on a Perkin Elmer Lambda 750 spectrometer where data were collected in 1mm path length cuvettes and were run versus the appropriate reference solvent. Static variable-temperature magnetic moment data were recorded in an applied dc field of 0.1 T on a Quantum Design MPMS XL7 superconducting quantum interference device (SQUID) magnetometer using doubly recrystallised powdered samples. Samples were carefully checked for purity and data reproducibility between several independently prepared batches for each compound examined. Data were measured on cooling and warming (average values presented), and samples were immobilised in an eicosane matrix to prevent sample reorientation during measurements. Diamagnetic corrections of were applied using tabulated Pascal constants and measurements were corrected for the effect of the blank sample holders (flame sealed Wilmad NMR tube and straw) and eicosane matrix. EPR spectra recorded on powdered samples (prepared likewise to the SQUID samples) were measured on a Bruker Elexsys E500 spectrometer. Cyclic voltammetry was carried out using a Metrohm AutoLab potentiostat controlled by NOVA. Under argon, a three-electrode configuration was employed with a platinum working electrode, a platinum wire secondary counter electrode, and a silver wire pseudo-reference electrode. Electrodes were polished using alumina/water prior to use. The THF solution was 1mM in test compound and 0.5

M in $[NBu_4][BF_4]$ as supporting electrolyte. Redox potentials are quoted versus the ferrocenium-ferrocene couple used as an internal reference by addition at the end of the experiment. Compensation for internal resistance was not applied. XANES samples were prepared under a $O_2/H_2O$ free atmosphere by mixing **3** and **4** with enough boron-nitride to provide samples with approximately 1000 ppm U. The resulting sample mixtures were pressed into a pellet and this was then placed in a double contained, airtight XAS sample cell. Standards ($[UI_3(THF)_4]$, nano-particulate $UO_2$, and $UO_2^{2+}$ sorbed onto ferrihydrite) were prepared under an oxygen-free atmosphere and packed in the same cells. The samples and standards were then stored frozen under argon prior to transport to Diamond Light Source, UK. The samples were transported to DLS on dry-ice in a sealed container that had been purged with argon. The samples were stored at DLS in a −20 °C freezer until analysis. The standards were stored at room temperature. The containers used for storage were purged with argon when removing samples or standards. XANES data were collected at 77 K in florescence mode using a 111 Si four-bounce monochromator and a 64 element monolithic Ge detector. Data were summed, normalised, and energy calibrated in Athena. XANES measurements at the Australian Synchrotron X-ray absorption spectroscopy beamline were performed with samples prepared as noted above. Conventional ion chambers and a 10×10 pixel array detector were utilized for transmission and fluorescence measurements, respectively. During the measurements, samples were maintained at a temperature of ~15 K. A $UO_2$ sample was measured simultaneously in transmission for energy calibration. Elemental microanalyses were carried out by Dr Tong Liu at The University of Nottingham, UK.

***Choice of Model Systems***

Geometry optimisations were only performed for the case where no ligand truncation is made, since the purpose of truncating the ligand was to study **3** and **4** where geometry optimisation is intractable for the full system. Therefore, for all model systems the truncations were made on the structure obtained by single crystal X-ray diffraction. Recall that in the experiments the $BIPM^R$ ligand ($BIPM^{TMS}$ =

$C(PPh_2NSiMe_3)_2$) is used. In the first model system, the phenyl rings are replaced with methyl groups (referred to as **2'**, **3'**, and **4'**; the ligand is denoted $BIPM^{L'}$ = $C(PMe_2NSiMe_3)_2$. On the other hand, the second truncation also replaces the $SiMe_3$ groups with $SiH_3$ (referred to as **2''**; the ligand is denoted $BIPM^{L''}$ = $C(PMe_2NSiH_3)_2$). Finally, the most significant truncation replaces both the $SiMe_3$ groups and phenyl groups with a hydrogen (referred to as **2'''** and **4'''**; the ligand is denoted $BIPM^{L'''}$ = $C(PH_2NH)_2$). The full ligand and all three truncations are studied for **2** with complete active space SCF with corrections to the energy from second-order perturbation theory (CASSCF/CASPT2).[3-5] The ring complex is only studied with RASSCF and CASPT2 for the smallest model, **4'''**; however, the largest models, **3'** and **4'**, are studied with density functional theory (DFT). See Figure S5 for a description of model systems and their labels.

***Density Functional Theory Calculations***

***Complex 2***

Geometry optimisations were performed for **2** at the PBE/def-TZVP[6,7] level of theory as implemented in the Turbomole software package.[8] For uranium and iodide, the associated small core def-ECP was used to account for scalar relativistic effects.[9] The resolution of the identity (RI) approximation was employed when computing the two-electron integrals.[7,10] The optimised geometry was confirmed as local minima by means of harmonic vibrational analysis. While lower spin states were also explored, only the high spin S = 2 state was optimised as lower spin states suffered from spin contamination.

***All Models***

Single point energy calculations were performed using the same level of theory as **2** for the high spin state for all of the models. High spin for the models of **2** is S = 2, while the high spin for models of **3** and **4** is S = 9/2. Natural population analysis was performed to compute charges and molecular orbitals were plotted and shown below for select systems (Figures S17 to S24)

***Complexes 3' and 4'***

Additionally, Nalewajski-Mrozek bond orders[11-15] and MDC-q charges [16]were calculated using the Amsterdam Density Functional (ADF) software package[17] with density functional theory (PBE/TZ2P)[18,19] for **3'** and **4'**. Note that for both sets of DFT calculations, only the high spin state is reported since lower spin states all suffered from spin contamination. A small core was used in the calculations along with the zero-order regular approximation (ZORA)[20-22] to include scalar relativistic effects.

***CASSCF/CASPT2 and RASSCF Calculations***

***Computational Details***

Cholesky decomposition and local exchange screening were used in all of the CASSCF/CASPT2 and RASSCF calculations to significantly reduce the cost of computing the two-electron integrals,[23-26] and scalar relativistic effects were taken into account through the use of the Douglas-Kroll-Hess Hamiltonian.[27-28] An imaginary shift 0.2 a.u and an IPEA shift of 0.25 a.u. were applied during the CASPT2 calculations. All calculations were performed using the Molcas program package.[29] Additionally, Mulliken charges are computed for all species, while Mulliken[30] and LoProp[31] charges are computed when the ANO-RCC[32,33] basis set is used. For complex **2** and its models, the S = 0, S = 1, and S = 2 states were explored. For **4''**, RASSCF calculations were performed for the S = 7/2 and S = 9/2 spin states. Lower spin states resulted in larger numbers of determinants and the wall time required for each SCF iteration was too demanding; however, CASPT2 calculations were performed on a smaller active space with (9*e*,11*o*) (only including the unpaired electrons in the 5f orbitals) for the S = 1/2 to the S = 9/2 spin states.

*Active Space Choice*

Perhaps the most important choice in a CASSCF calculation is the active space. Ideally, all of the valence orbitals would be included; however, in most cases the inclusion of ligand orbitals in the active space is not required to properly describe the ground state of organometallic compounds. For actinide-containing systems in higher oxidation states, only the 5*f* orbitals on the uranium centre must be included in the active space (*e.g.* including the 6*d* and 7*s* orbitals is not required).[34] Therefore, the best active space for **2** includes molecular orbitals that are linear combinations of the 5*f* orbitals on both metal centres resulting in an active space of eight electrons in fourteen orbitals, denoted (8*e*,14*o*). This would translate to an active space with 42 orbitals for **3** and **4**, far beyond the limits of this method. Therefore, we explored smaller active spaces to determine the minimum space required to best address our initial question of the nature of bonding within the U-arene-U group and the number of unpaired electrons in each arene group in **3** and **4**. By removing orbitals with occupation numbers less than 0.03 from the (8*e*,14*o*) active space for **2**, an active space of eight electrons in eight orbitals, (8*e*,8*o*), was identified and tested for **2**. This smaller active space yields results consistent with the best active space of (8*e*,14*o*) (see Table S3).

Furthermore, **3** and **4** contain three U-arene-U group, tripling the size of the required active space compared to **2**. Our testing in **2** indicates that no fewer than (8*e*,8*o*) per arene should be used in the ring complexes. Therefore, an initial guess at the best active space would be to include the 24 orbitals analogous to those in **2**. However, recall that the ligand has been deprotonated in **3** and **4**. As a result, there are fewer electrons associated with each U-arene-U group leading to an analogous active space of 21 electrons in 23 orbitals (21*e*, 23*o*). This active space is still too large to be treated with CASSCF; however, the RASSCF approach can be used. The RASSCF calculations use an active space of (21*e*,2*e*,2*e*;6*o*,11*o*,6*o*) where the notation of Sauri *et al.*[35] indicates a RAS space of (n,l,m;i,j,k) where n is the number of electrons in the active space, l is the maximum number of holes in RAS1, and m is the

number of electrons allowed in RAS3. Similarly, i, j, and k are the number of orbitals in RAS1, RAS2, and RAS3 respectively. Calculations on **4''** were performed with $C_2$ symmetry, while all other calculations were in $C_1$. RAS1 includes the δ-bonding orbitals, RAS2 includes the orbitals containing the unpaired electrons, and RAS3 includes the δ* orbitals.

***Complexes 2, 2', 2'', and 2'''***

The electronic structure of **2** and its models were explored using CASSCF/CASPT2 calculations. The ANO-RCC basis set was employed for uranium atoms using a contraction of triple-ζ quality, while the N, P, and $C_{arene}$ and $H_{arene}$ atoms were treated with a basis set of double- ζ quality and peripheral I, Si, C, and H atoms were treated with a minimal basis set.[32,33] The (8*e*,14*o*) and (8*e*,8*o*) active spaces were computed for **2** and the three models.

***Complexes 2', 2'', 2''', and 4'''***

For the models of **2**, CASPT2 calculations were performed using the (8*e*,8*o*) active space and the smaller basis set that will also be employed in calculations of **4'''** to assess not only the effect of truncating the active space but also the effect of using a small basis set. The Dolg ECP basis set[9] was used for all atoms except for H where the 3-21G basis set was employed. For uranium, the small core ECP was chosen. Finally, for **4''**, a larger active space is required and therefore RASSCF calculations were performed; however, the ECP basis set is also used.

**Supplementary Note 1**

***DFT and CASPT2 Results for 2***

The PBE/def-TZVP geometry optimisation of **2** for the high spin S = 2 state gave structures in good agreement with experiment and previous density functional theory results (Table S1). For the optimised geometries, DFT single-points were performed for the S = 0 and S = 1 states. As expected, the closed shell singlet is high in energy; therefore, the broken symmetry singlet is also computed. Both the broken symmetry singlet and the triplet are higher in energy than the quintet state and have some spin contamination (Table S2). CASPT2 calculations were also performed and the results are given in Table S3. Our best calculation used the ANO-RCC basis sets and the (8*e*,14*o*) active space. The bonding in **2** has four singly occupied natural orbitals composed of linear combinations of 5*f* orbitals localised on the uranium centres (Figure S1). The two δ−bonding orbitals in **2** have occupation numbers of 1.85 and 1.86. This is consistent with the DFT study previously published concurrently with the synthesis of this molecule. The S = 0, 1, and 2 states are computed, where the singlet is the lowest in energy and the triplet lies only 0.03 kcal/mol higher (Table S3). The quintet state is also relatively close in energy at 2.41 kcal/mol. Each uranium center has an electron configuration of $5f^2$ and was previously assigned as having two uranium(III) centers.

The (8*e*,14*o*) active space is compared with the (*8e,8o*) active space for **2** (see Table S3). Despite reducing the size of the active space, the overall picture remains the same but the occupation numbers of the δ−bonds increase from 1.85-1.87 to 1.94-1.95. Additionally, the relative energies at the CASPT2 level with the (8*e*,14*o*) space and those with the (*8e,8o*) space differ by less than 0.5 kcal/mol, well within the error of the method. The CASSCF energies were more sensitive than the CASPT2 energies but for this system the PT2 part can recover the energy missing by using the smaller active space. For these reasons, the (*8e,8o*) space is suitable for use in describing the ground state of **2**. Additionally,

Mulliken and LoProp charge analysis were performed on these compounds (Table S4) and reducing the active space does not qualitatively change the charges. Note that the Mulliken charges have a very low charge on the bridging arene compared to the LoProp charge.

***Effect of Ligand Truncation in 2***

The most significant approximation we make is the truncation of the BIPM ligand. In approaching this rather severe ligand truncation, there was the possibility that this could have serious implications in our calculations. Therefore, we first apply the ligand truncations to **2** to test if 1) the ligand truncation leads to qualitatively different results and 2) that truncating the active space to (8*e*,8*o*) has a minimal effect regardless of the model. In Table S5, the CASSCF and CASPT2 energies are reported for **2** and the three model systems for both the (8*e*,14*o*) and the (8*e*,8*o*) active spaces. Comparing **2** to the smallest model **2'''**, with the larger active space the difference in CASPT2 energies between the singlet and quintet states changes from 2.41 for **2** to 3.62 for **2'''**. This is a change in 1.21 kcal/mol. Given the large ligand truncation, we consider this effect rather small in that the results are qualitatively the same. Additionally, the change from the larger to the smaller active space is again less than 0.3 kcal/mol for all models. Additionally, the bonding picture remains qualitatively unchanged with respect to the composition of the natural orbitals and occupation numbers. The CASSCF natural orbitals and their occupation numbers are given in Figures S8 to S13. Again, we compute the LoProp and Mulliken charges. As was observed in **2**, the largest difference between LoProp and Mulliken arises in the total charge on the bridging toluene group. As the model gets smaller, the Mulliken charges are larger. This is not particularly surprising since a known failure of Mulliken charges is their sensitivity to the number of basis functions. Unlike in **2** where the Mulliken charge on uranium and the methanide carbon were quite similar to the LoProp charge, the Mulliken charges differ from the LoProp charges to a larger extent when the ligand is truncated. Again, we recommend that the reader consider Mulliken

charges as only a qualitative guide given their well-known deficiencies. We do note that both the LoProp and Mulliken chargers are not effected by truncating the active space.

### *CASPT2 Results for 2 and its Models with a Smaller Basis Set*

Next, results from the calculations with the ECP basis set are compared to those using ANO-RCC. In this case, only the (8*e*,8*o*) active space was computed since for **4''** neither the ANO-RCC basis set nor such a large active space will be applied. In Table S7, the relative energies are again in agreement by less than 0.5 kcal/mol. The active orbitals and occupation numbers (Figures S14 to S16) are the same for the ECP basis as they were with ANO-RCC when the same active space is used. While it is well known that Mulliken charges are sensitive to the basis set (and we certainly see this in our results), it is important to emphasise that the trends between the charges for the charge models are consistent whether the ANO-RCC or ECP basis set is used. For example, in Table S8, the charges for the system with the largest number of basis functions, **2**, has LoProp charges that compare well with the other models; however, the Mulliken charges on the arene group are very small in **2** and we attribute this to the well-known problems associated with Mulliken charges. By studying **2** and its representative model systems, three main conclusions arise: 1) the (8e,8o) active space is large enough to properly describe the ground state; 2) the Dolg ECP basis set gives results in qualitative agreement with the ANO-RCC basis; 3) truncating the ligand does not change the nature of the ground state qualitatively.

### *RASSCF Results for 4'''*

Relative energies with RASSCF for **4''** are given in Table S9. Since RASPT2 calculations with the large basis set required more memory than was feasible on our resources, only RASSCF energies are reported for our largest active space. However, a smaller active space can be employed in which the δ-bonding orbitals with the highest occupation numbers (and their corresponding anti-bonding orbitals) were removed from the active space. This (9*e*,17*o*) active space can be used to perform CASPT2

calculations. While this is an approximation, we can use this smaller active space to compute CASPT2 energies (see Table S10). Note that the figures of the active orbitals for RASSCF were reported in the main text.

### ***DFT Results for 2 and its Models***

PBE/def-TZVP calculations were performed for **2** and its three models for the high spin state only. Natural population analysis (NPA) as implemented in the Turbomole program package was performed to compute charges.

### ***DFT Results for 3' and 4' and its Models***

Finally, DFT calculations were performed for **3'**, **4'**, and **4'''**. Furthermore, no symmetry was imposed in the DFT calculations whereas the RASSCF calculations were performed in $C_2$ symmetry. The δ and 5f orbitals are drawn in Figure S21 and S23 while the orbitals with contributions from the 2p orbitals on the carbene are plotted in Figures S22 and S24 for **3'** and **4'**, respectively. Charge analysis was performed with natural population analysis (NPA) using density functional theory (PBE/def-TZVP). The charges on uranium are lower than with the other approaches and once again we see that changing the charge model has a larger effect than the ligand truncation. Additionally, since previous DFT studies by some of the authors have used DFT to compute MDC-q charges as implemented in the ADF program package, single point energy calculations were performed for **3'** and **4'** for comparison with the literature.

## Supplementary References


1. Mills, D. P. *et al. Nat. Chem.* **3**, 454-460 (2011).
2. Bergbreiter, D. E. & Killough, J. M. *J. Am. Chem. Soc.* **100**, 2126-2134 (1978).
3. Roos, B. O. & Taylor, P. R. *Chem. Phys.* **48**, 157-173 (1980).
4. Andersson, K., Malmqvist, P. A. & Roos, B. O. *J. Chem. Phys.* **96**, 1218-1226 (1992).
5. Andersson, K., Malmqvist, P. A., Roos, B. O., Sadlej, A. J. & Wolinski, K. *J. Phys. Chem.* **94**, 5483-5488 (1990).
6. Perdew, J. P., Burke, K. & Ernzerhof, M. *Phys. Rev. Lett.* **77**, 3865-3868 (1996).
7. Eichkorn, K., Weigend, F., Treutler, O. & Ahlrichs, R. *Theor. Chem. Acc*. **97**, 119-124 (1997).
8. TURBOMOLE V6.2 2010, a development of University of Karlsruhe and Forschungszentrum Karlsruhe GmbH, 1989-2007, TURBOMOLE GmbH, since 2007, available from http://www.turbomole.com.
9. Cao, X., Dolg, M. & Stoll, H. *J. Chem. Phys.* **118**, 487-496 (2003).
10. Eichkorn, K., Treutler, O., Ohm, H., Haser, M. & Ahlrichs, R. *Chem. Phys. Lett.* **240**, 283-289 (1995).
11. Michalak, A., DeKock, R. L. & Ziegler, T. J. *Phys. Chem. A* **112**, 7256-7263 (2008).
12. Nalewajski, R. F. & Mrozek, J. *Int. J. Quant. Chem*. **51**, 187-200 (1994).
13. Nalewajski, R. F. & Mrozek, J. *Int. J. Quant. Chem*. **61**, 589-601 (1997).
14. Nalewajski, R. F., Mrozek, J. & Michalak, A. *Polym. J. Chem*. **72**, 1779-1791 (1998).
15. Nalewajski, R. F., Mrozek, J. & Mazur, G. *Can. J. Chem*. **74**, 1121-1130 (1996).
16. Swart, M., Van Duijnen, P. Th. & Snijders, J. G. *J. Comp. Chem*. **22**, 79-88 (2001).
17. Te Velde, G., *et al*. *J. Comp. Chem.* **22**, 931-967 (2001).
18. Perdew, J. P., Burke, K. & Ernzerhof, M. *Phys. Rev. Lett.* **77**, 3865-3868 (1996).
19. Van Lenthe E. & Baerends, E. J. *J. Comp. Chem.* **24**, 1142-1156 (2003).
20. Van Lenthe, E., Baerends, E. J. & Snijders, J. G. *J. Chem. Phys***.** **99**, 4597-4610 (1993).

21. Van Lenthe, E., Baerends, E. J. & Snijders, J. G. *J. Chem. Phys*. **101**, 9783-9792 (1994).

22. Van Lenthe, E., Ehlers, A. E. & Baerends, E. J. *J. Chem. Phys*. **110**, 8943-8953 (1999).

23. Aquilante, F., Gagliardi, L., Pedersen, T. B. & Lindh, R. *J. Chem. Phys.* **130**, 154107 (2009).

24. Aquilante, F., Lindh, R. & Pedersen, T. B. *J. Chem. Phys.* **129**, 034106 (2008).

25. Aquilante, F., Malmqvist, P. A., Pedersen, T. B., Ghosh, A. & Roos, B. O. *J. Chem. Theo. Comput.* **4**, 694-702 (2008).

26. Aquilante, F., Pedersen, T. B. & Lindh, R. *J. Chem. Phys.* **126**, 194106 (2007).

27. Douglas, M. & Kroll, N. M. *Annals of Physics* **82**, 89-155 (1974).

28. Hess, B. A. *Phys. Rev. A* **33**, 3742-3748 (1986).

29. Aquilante, F. *et al*. *J. Comp. Chem.* **31**, 224-247 (2010).

30. Mulliken, R. *Int. J. Chem. Phys*. **23**, 1833−1840 (1955).

31. Gagliardi, L., Lindh, R. & Karlström, G. *J. Chem. Phys*. **121**, 4494−4500 (2004).

32. Roos, B. O., Lindh, R., Malmqvist, P. A., Veryazov, V. & Widmark, P. O. *J. Phys. Chem. A* **108**, 2851-2858 (2004).

33. Roos, B. O., Lindh, R., Malmqvist, P. A., Veryazov, V. & Widmark, P. O. *Chem. Phys. Lett.* **409**, 295-299 (2005).

34. Veryazov, V. *et al*. *Int*. *J*. *Quant*. *Chem*. **111**, 3329-3338 (2011).

35. Sauri, V. *et al*. *J. Chem. Theory Comput.* **7**, 153–168 (2011).
